# Use of ^31^P magnetisation transfer magnetic resonance spectroscopy to measure ATP changes after 670 nm transcranial photobiomodulation in older adults

**DOI:** 10.1111/acel.14005

**Published:** 2023-10-06

**Authors:** Elizabeth J. Fear, Frida H. Torkelsen, Elisa Zamboni, Kuan‐Ju Chen, Martin Scott, Glenn Jeffery, Heidi Baseler, Aneurin J. Kennerley

**Affiliations:** ^1^ Hull York Medical School University of York York UK; ^2^ Department of Biomolecular Sciences University of Urbino Carlo Bo Urbino Italy; ^3^ Department of Chemistry University of York York UK; ^4^ Department of Psychology University of York York UK; ^5^ School of Psychology University of Nottingham Nottingham UK; ^6^ Department of Psychology Stanford University Stanford California USA; ^7^ Faculty of Brain Sciences Institute of Ophthalmology, UCL London UK; ^8^ Institute of Sport Manchester Metropolitan University Manchester UK

**Keywords:** ^31^P magnetic resonance spectroscopy, ageing brain, cell metabolism, magnetisation transfer, photobiomodulation

## Abstract

Mitochondrial function declines with age, and many pathological processes in neurodegenerative diseases stem from this dysfunction when mitochondria fail to produce the necessary energy required. Photobiomodulation (PBM), long‐wavelength light therapy, has been shown to rescue mitochondrial function in animal models and improve human health, but clinical uptake is limited due to uncertainty around efficacy and the mechanisms responsible. Using ^31^P magnetisation transfer magnetic resonance spectroscopy (MT‐MRS) we quantify, for the first time, the effects of 670 nm PBM treatment on healthy ageing human brains. We find a significant increase in the rate of ATP synthase flux in the brain after PBM in a cohort of older adults. Our study provides initial evidence of PBM therapeutic efficacy for improving mitochondrial function and restoring ATP flux with age, but recognises that wider studies are now required to confirm any resultant cognitive benefits.

## Graphical abstract

Mitochondrial function, and so ATP production, declines with age. We use ^31^P magnetic resonance spectroscopy, in a cohort of older adults to quantify increases in the rate of ATP synthase flux in the brain following 670 nm photobiomodulation treatment. Our study provides initial evidence of PBM therapeutic efficacy for improving mitochondrial function and restoring ATP flux with age.

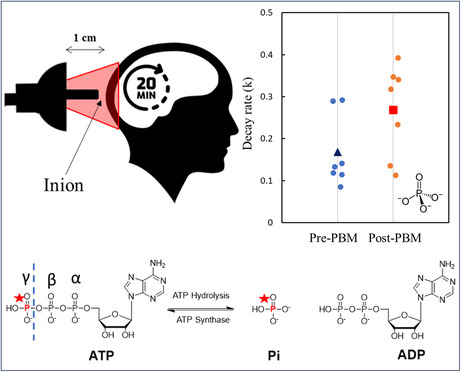

**Abbreviations**
ADP: adenosine diphosphate
ATP: adenosine triphosphate
BW: bandwidth
Cr: creatine
ETC: electron transport chain
FWHM: full width at half maximum
MCS: Monte Carlo simulation
MRS: magnetic resonance spectroscopy
MT: magnetisation transfer
PBM: photobiomodulation
PCr: phosphocreatine
Pi: inorganic phosphate
RF: radiofrequency
ROS: reactive oxygen species
TE: echo time
TR: repetition time

## 1 INTRODUCTION

**FIGURE 1:**
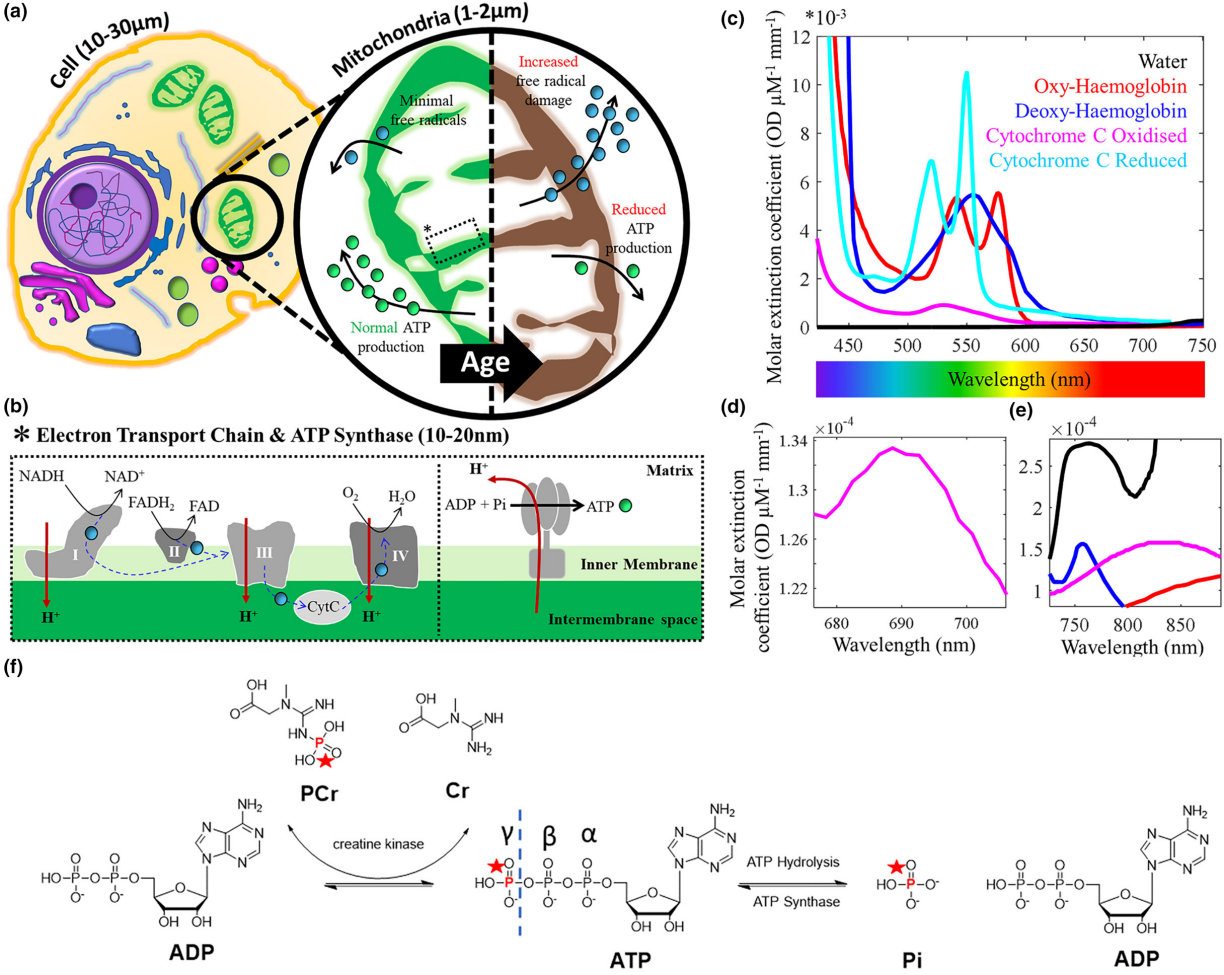
Key mediators of ATP production in the mammalian cell. (a) Aerobic metabolism occurs in mitochondria and drives ATP (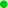) production. With age, chronic exposure of mitochondrial membranes to reactive oxygen species (ROS, 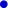), released a consequence of metabolism/other, are hypothesised to drive decreases in cell function and viability (Balaban et al., 2005; Guo et al., 2013). ATP production reduces & can accelerate age‐related neurodegenerative conditions (Kasote et al., 2013); (b) ROS are utilised as part of the ETC to alter membrane potential. A key mediator to help passage ROS through the various protein complexes (specifically III and IV) is cytochrome c. Movement of protons back into the mitochondrial matrix via ATP synthase produces ATP from ADP and inorganic phosphate (Pi). “Activating” cytochrome c & cytochrome c oxidase (complex IV) with light is hypothesised to “maintain” the ETC process and so slow the effects of ageing. (c) Cytochrome is a key chromophore with molar extinction coefficients similar to haemoglobin; (d) The oxidised state of cytochrome c has a small peak in absorption at 695 nm due to the haem iron‐Met80 bond; (e) Copper centres in cytochrome c oxidase have a broad absorption range that peaks at ~830 nm. These near‐infrared photon wavelengths can penetrate the skin/skull into brain tissue for activation of cytochrome c. (f) ATP is mainly generated through oxidative phosphorylation in the mitochondria, and glycolysis in the cytosol. Redox reactions, as part of the ETC, transfer electrons from nicotinamide and flavin adenine dinucleotide mediators (NADH & FADH_2,_ respectively), to oxygen. Resultant energy is used to pump H^+^ from the mitochondrial matrix to the intermembrane space, creating an electrochemical gradient driving the synthesis of ATP from ADP and P_i_, via ATP synthase. ATP hydrolysis (i.e., use and breakdown back into ADP and P_i_) is high as ATP is required in many cellular processes (Zhu et al., 2012). Creatine kinase (CK) in the cell cytoplasm also maintains the homeostasis of cellular energy. A reservoir of PCr is maintained at a concentration much higher than that of ATP. This makes it possible to carry out many energy‐draining tasks quickly (Schlattner et al., 2016).

Mitochondrial function declines with age due to time‐related oxidative damage, cysteine toxicity, mitochondrial DNA (mtDNA) mutations (either inherited or somatic) (Balaban et al., 2005; Guo et al., 2013; Kasote et al., 2013; Mammucari & Rizzuto, 2010), and impaired biogenesis (Ames et al., 1995; Conley et al., 2007; Hughes et al., 2020; Kokoszka et al., 2001; Markaki & Tavernarakis, 2020; McGuire, 2019). Neurological/psychological conditions (e.g., brain injury, stroke, Alzheimer's disease, Parkinson's disease, depression, anxiety, and age‐related cognitive decline) further render neuronal mitochondria vulnerable to oxidative stress (Fujimura et al., 1998; Rabuffetti et al., 2000; Sugawara et al., 1999). Alteration of the electron transport chain (ETC) ultimately reduces adenosine triphosphate (ATP) production and, in turn, increases apoptosis (Figure 1a). Therefore, an ageing population coupled with associated increases in cases of neurological conditions (Bejot & Yaffe, 2019) amplifies the need to develop safe, inexpensive treatments to restore mitochondrial function and offer neuronal protection as we grow old. Evidence shows that *non‐invasive* transcranial red/infrared photobiomodulation (PBM) therapy can offer such neuroprotective benefits (Hamblin, 2016, 2018).

PBM illuminates the tissue with narrowband light in the 600–1100 nm wavelength range (Chung et al., 2012; Salehpour et al., 2018). Reported benefits of PBM are wide reaching. In preclinical models PBM has been shown to improve inflammatory arthritis (Castano et al., 2007), aid wound healing (Conlan et al., 1996), stimulate muscle regeneration (Oron, 2006), and reduce necrosis in ischemic heart muscle (Oron et al., 2001). Improvements in sleep quality, mood, and cognitive function have also been observed as a consequence of PBM therapy applied to the brain through the nasal cavity or via custom light helmets (Berman et al., 2017; Saltmarche et al., 2017). Recent studies have used PBM to reduce the severity of COVID‐19 (Fernandes et al., 2020; Liebert et al., 2020).

In terms of neuroprotection, transcranial PBM (usually in the near‐infrared spectrum to penetrate through the scalp and skull) has been used to reduce damage caused by stroke (ischemic or other) (Lapchak et al., 2004; Yip et al., 2011) and remarkably, has been shown to induce neurogenesis (Lampl et al., 2007; Oron et al., 2006; Zivin et al., 2009). Marked neurological improvements in Alzheimer's disease (De Taboada et al., 2011; Johnstone et al., 2016), Parkinson's disease (Johnstone et al., 2016; Liebert et al., 2021; Maloney et al., 2010; Oueslati et al., 2015; Zhao et al., 2003), depression and anxiety (Salehpour & Rasta, 2017; Schiffer et al., 2009), traumatic brain injury (Ando et al., 2011) retinal ageing and disease (Begum et al., 2013; Gkotsi et al., 2014; Kaynezhad et al., 2021; Muste et al., 2021; Shinhmar et al., 2020), and general decelerated cognitive decline in ageing (Salehpour et al., 2017) have all been documented.

While the benefits of PBM are widely reported, controversy remains over the exact mechanism responsible for the observed positive effects. This limits clinical recognition of PBM therapy. We test the hypothesis that PBM therapy yields an increased rate of ATP production. Animal models of ageing and neurodegenerative disease have shown that PBM application improves mitochondrial membrane potential and associated ATP production (Begum et al., 2013; Gkotsi et al., 2014; Kam et al., 2021; Kaynezhad et al., 2021; Shinhmar et al., 2020). Here we use magnetisation transfer (MT)‐based ^31^P magnetic resonance spectroscopy (MRS) to quantify, for the first time, the ATP production rate pre‐ and post‐670 nm PBM (Hoult et al., 1974; Ingwall, 1982; Liu et al., 2017) of the ageing human brain (60 years+). ^31^P‐MRS is a non‐invasive method for measuring “relative” intracellular concentrations of phosphorus‐containing metabolites including ATP, inorganic phosphate (Pi—used in anaerobic glycolysis and aerobic oxidative phosphorylation in the mitochondria via ATP synthase), and phosphocreatine (PCr—used in anaerobic creatine kinase pathways to supplement ATP production during energy‐draining tasks (Schlattner et al., 2016; Zhu et al., 2012)). These important metabolites are identifiable through their differences in MR chemical shift. Magnetic labelling of the terminal phosphate (γ) of ATP with a radio frequency (RF) pulse train induces a signal decrease (via MT) in both of its exchange partners (Pi and PCr). By varying the pulse train length, one can estimate the forward exchange rate of ATP for both pathways. Key reaction kinetics relating to ATP production (creatine kinase & ATP synthesis/hydrolysis cycle bioenergetic reactions) can therefore be probed non‐invasively with MT‐MRS (Bittl & Ingwall, 1985).

### 1.1 Mechanisms of PBM action

The leading hypothesis about the mechanism of PBM therapeutics involves mitochondrial homeostasis. Within mitochondria, the balance between reactive oxygen species (ROS) and ATP production is preserved by integrating multiple cellular signals (Jezek & Hlavata, 2005; Samavati et al., 2008). When too many ROS are produced, major disturbances in cell function and viability occur, leading to disease (Figure 1a) (Duvigneau et al., 2008; Huettemann et al., 2011; Kadenbach, 2018; Samavati et al., 2008).

The inner mitochondrial membrane houses carrier proteins (complexes I‐IV) responsible for the induction of an electrochemical proton gradient to drive ATP synthase (as part of the ETC—Figure 1b). The enzyme cytochrome c oxidase (CCO), known as complex IV, catalyses the rate‐determining step in the ETC (Villani & Attardi, 1997). CCO is also involved in the formation of apoptosome, and therefore, the progression of apoptosis (Huettemann et al., 2011). CCO is a photoreceptor with a broad absorption spectra (Figure 1c). Within oxidised CCO, the haem iron‐Met80 bond causes increased light absorption at ~695 nm (Figure 1d) (Huettemann et al., 2011). The copper centres in CCO have a broader absorption range that peaks around 830 nm (Figure 1e) (Jagdeo et al., 2012). It is hypothesised that photoexcitation of the electronic states of these compounds changes the redox properties, which are fundamental for the effective generation of the electrochemical membrane proton gradient driving ATP synthase as part of the ETC (Karu, 1999). Indeed, Wong‐Riley et al. (2005) illuminated primary neurons (with 670, 728, 770, 830, and 880 nm light) functionally inactivated by toxins (Wong‐Riley et al., 2005). The greatest increase in energy metabolism occurred under 830 nm and 670 nm light. The least effective wavelength was found to be 728 nm. This was attributed as evidence for the upregulation of CCO. An array of in vitro cell culture data now exists, confirming increases in ATP synthesis following PBM (Mochizuki‐Oda et al., 2002; Oron et al., 2007; Yu et al., 1997). Furthermore, any absence of these near‐infrared absorption bands implies a dysfunctional/denatured conformation of CCO and is often referred to as an “indicator of trouble”. Keeping CCO upregulated with photoexcitation was hypothesised to inhibit release into the cytoplasm and stave off cell death/ageing.

However, maximum light absorbance for CCO occurs at <425 nm (Figure 1c) (Mason et al., 2014). Therefore, it would be expected that the ATP production rate would be superior, illuminating cells at this wavelength of light. Counterintuitively, when human adipose stem cells were exposed to blue light (415 nm, 16 mW cm^−2^), a reduction of intracellular ATP levels was observed (with an increase in intracellular ROS). Longer exposure to this blue light resulted in further decreases in ATP levels (Kam et al., 2021; Kaynezhad et al., 2022; Mason et al., 2014). Alternative mechanisms involving a reduction in interfacial water layer (IWL) viscosity in the irradiated cells were therefore proposed (Butt & Keilin, 1962; Sommer, 2019).

The beneficial effects of infrared based PBM could also be caused by photo based generation of singlet oxygen (particularly at long wavelength 1064/1280 nm illumination), localised transient heating of absorbing chromophores, and increased superoxide anion production with subsequent increase in concentration of the product of its dismutation, H_2_O_2_ (Karu, 1999). Nitric oxide (NO) is proposed to be released upon cell illumination (photo‐dissociation of NO from the binuclear centre (a_3_/Cu_B_) of CCO) (Salehpour et al., 2018), which causes vasodilation and therefore increased blood flow to the vicinity of the cell. While such vasodilation would deliver more glucose and oxygen for increased metabolism, it could also act as a heat sink and mitigate the tissue heating effects of direct illumination. Indeed, recent studies have attempted to model bio‐heat transfer in the human brain. While the scalp, under direct 20‐min illumination, can increase in temperature by 0.5°C, initial simulations note minor changes in cerebral cortex temperature <0.02°C (Ibrahimi & Delrobaei, 2022). Whatever the direct mechanism, these systemic effects have been reported to be short‐term (i.e., occurring during PBM treatment (Hamblin, 2016; Khan et al., 2015; Mitrofanis, 2017; Rojas & Gonzalez‐Lima, 2011)) and warrant further time‐related simulations.

Formation of ROS after CCO excitation has also been suggested as a mediator of the beneficial biological effects of PBM (780 nm illumination) therapy (Grossman et al., 1998). ROS are important in the activation of transcription factors in the nucleus. These transcription factors lead to the upregulation of stimulatory and protective genes leading to cell proliferation/neurogenesis (Zhang et al., 2003). Importantly such effects have been found to continue after PBM treatment and may explain the positive effects found days, weeks, and months after treatment (Hamblin, 2016; Mitrofanis, 2017; Rojas & Gonzalez‐Lima, 2011).

## 2 MATERIALS AND METHODS

### 2.1 Participant information

Written informed consent for this study was obtained from all participants. Ethical approval was granted by the York Neuroimaging Centre (YNiC) Research, Ethics, and Governance Committee following the tenets of the Declaration of Helsinki. Ten healthy participants aged 60+ were recruited from the YNiC volunteer list (mean age = 68 years; age range = 60–85 years; 6 females, sex assigned at birth). General observations regarding hair colour, hair coverage, and skin tone (all participants were white British) were made and informed statistical models (Table S1). Volunteers were excluded if they had contraindications for completing MRI procedures, known neurodegenerative conditions, were currently enrolled in an interventional clinical trial, and/or if they were unable to comply with the study.

### 2.2 Experimental design

**FIGURE 2:**
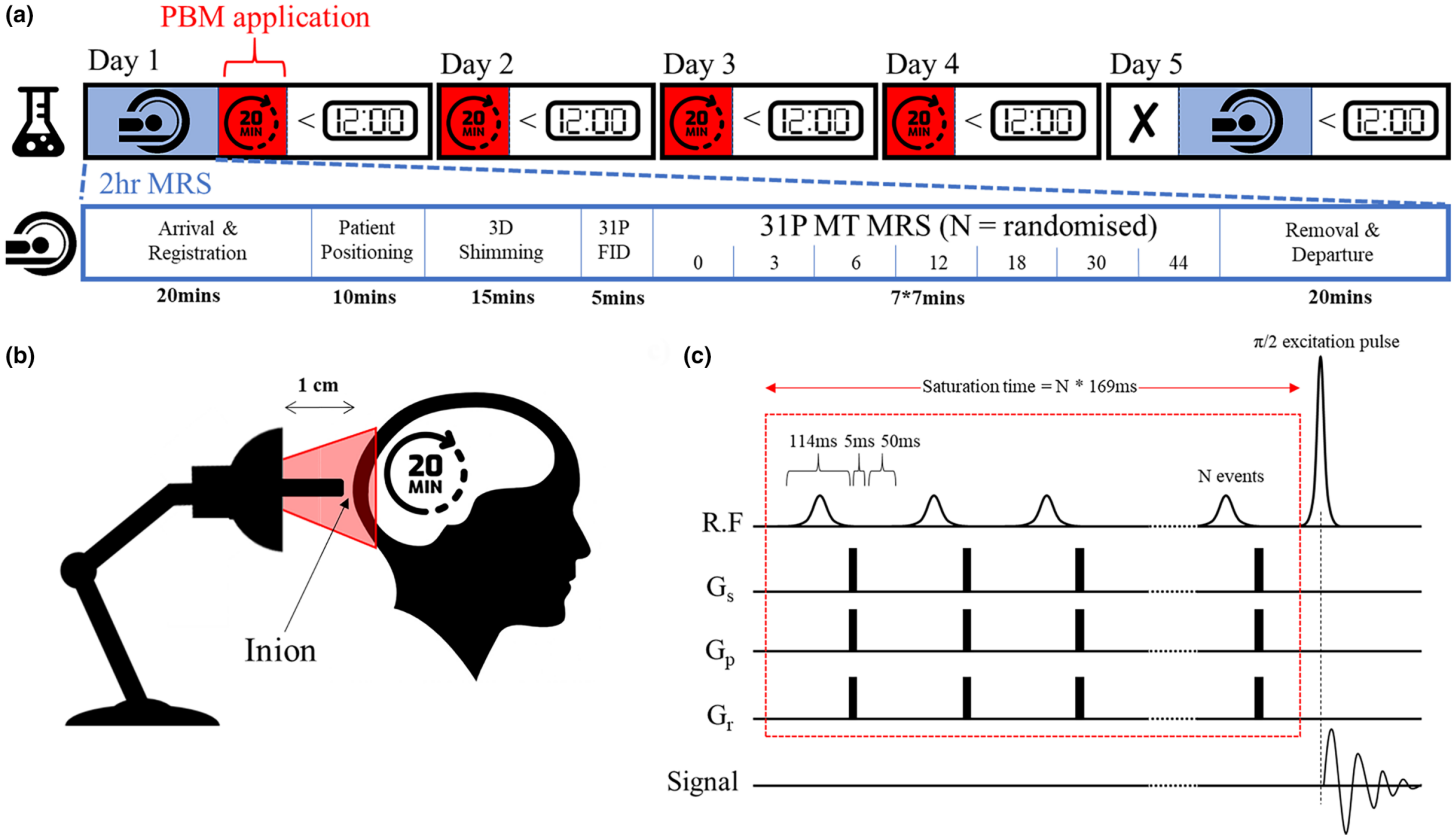
Experimental design for tracking the neuroprotective effects of PBM treatment. (a) Day 1: Baseline ^31^P MRS measures followed by 20 min PBM (670 nm LED bulb) applied ~1 cm from the inion (b). PBM repeated for 3 days before midday. Day 5: post‐MRS measurements (N.B. no PBM applied on this day); (c) ^31^P MRS utilised a MT FID‐based appraoch with a varying number of 114 ms saturation RF pulses (0, 3, 6, 12, 18, 30, and 44) followed by a 5 ms spoiler gradient and 50 ms delay. Experiment order randomised for each scanning session.

A 5‐day longitudinal study was designed (Figure 2a). Baseline ^31^P MT‐MRS assessments were completed on the morning of Day 1. Immediately after scanning, participants underwent their first session of PBM and training for self‐application for the next 3 days. PBM was applied before midday over a 4‐day period in accordance with data from Weinrich et al. (2019) and Shinhmar et al. (2021). On Day 5, participants completed a post‐^31^P MT‐MRS assessment to investigate changes in ATP flux. We constructed this 5‐day experimental design based on Wong‐Riley et al. (2005), who showed that LED treatment at 670 nm significantly reversed the detrimental effect of a toxin on neurons using a similar cumulative dosage (*p* < 0.001 for three metabolic neuronal types) (Wong‐Riley et al., 2005).

PBM treatment used a narrowband (peaking at 670 nm) E27 Edison screw (27 mm diameter) flat‐ended light bulb consisting of 12 LED sources with a 30‐degree beam angle (18W, Red Mini 670, Red Light Man Ltd., Manchester, UK), mounted on a spring‐loaded (angle‐poise) adjustable arm. This allowed the participant easy placement while sat/positioned comfortably. The centre of the bulb was held 1 cm from the participant's inion using a small spacer (Figure 2b). PBM therapy targets the occipital lobes of the human brain. The comparatively high concentration of metabolites found in the brain, which are often altered in neurodegenerative diseases, can be exploited (Duarte et al., 2012). The occipital lobes were selected because of their ease of access, density of grey matter, and proximity to the MRS surface coil used for measurement in the scanner to ensure patient comfort in a supine position. The light was administered for 20 min (participants were supplied with a digital timer). Participants were instructed to sit as still as possible during PBM application but to immediately stop application if the scalp felt uncomfortably warm. For standardised reporting of treatment, see tables in Appendix S1 (Jenkins & Carroll, 2011).

#### 2.2.1 Magnetic resonance spectroscopy

MRS measurements utilised a 3 T MAGNETOM Prisma system (Siemens Healthcare GmbH, Erlangen, Germany). A dual‐channel ^1^H/^31^P transmit‐receive flexible surface coil (O‐XL‐HL‐030‐01150 V03; Rapid Biomedical GmbH, Rimpar, Germnay) was used for RF transmission/detection. The coil was positioned at the head of the scanner bed, and participants were placed in a supine position. The inion was positioned over the centre of the flex surface coil, and foam cushioning was used to limit lateral bending and aid comfort. Magnet‐safe padding was used to curve the flexible coil on either side of the head. Participants were instructed to stay as still as possible during measurements. No head restraints were used, and participants were allowed to listen to music while in the scanner. Participants were given foam ear plugs to protect their hearing during scans.

**FIGURE 3:**
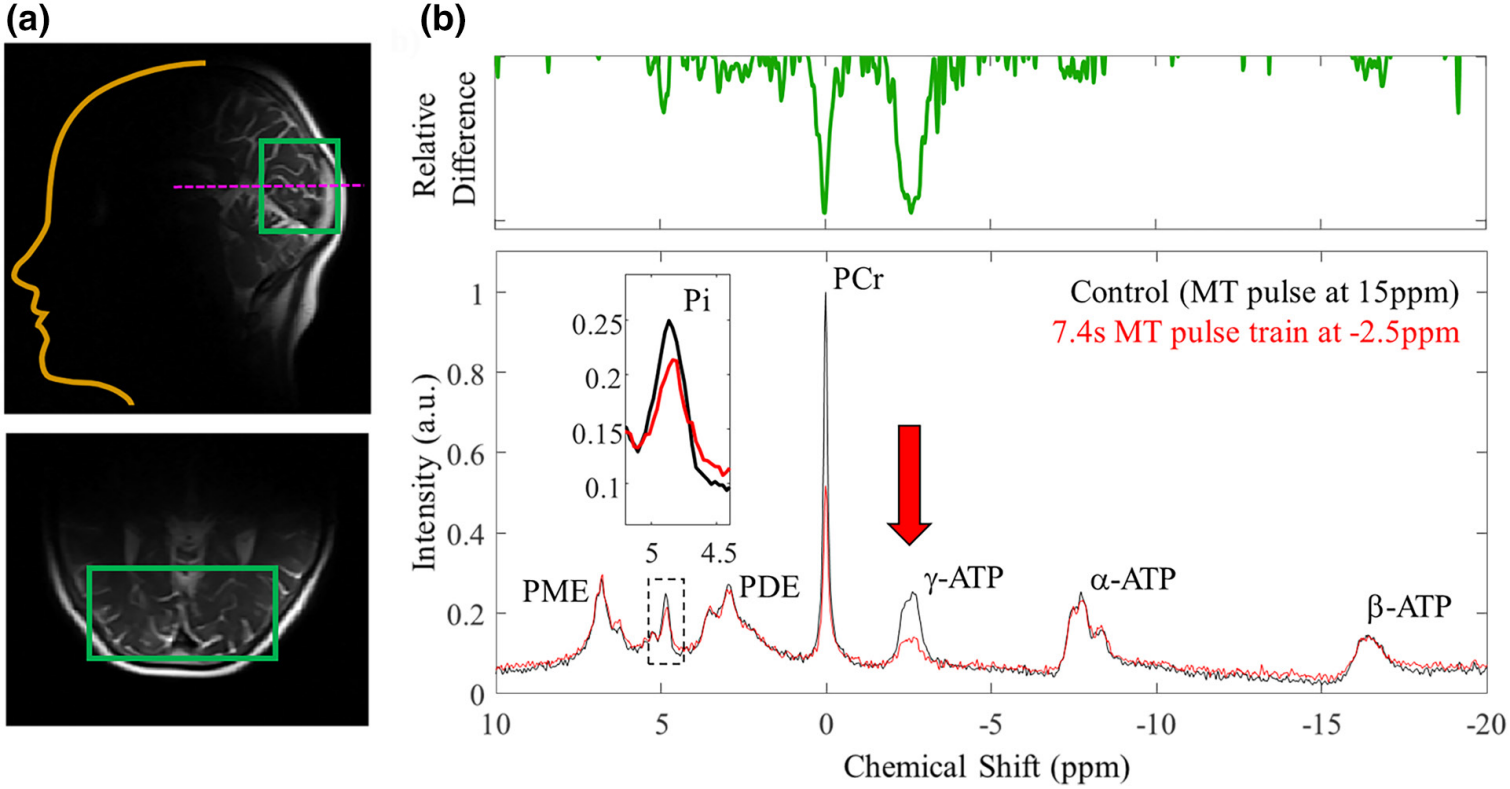
Representative ^31^P MR spectra. (a) T_2_ localiser used to position adjustment window covering the visual cortex. Post frequency matching, power calibration, and shimming adjustments, ^31^P spectra were acquired. (b) Phased & baseline corrected ^31^P spectra (40 ppm spectral width) used to identify key metabolites including adenosine triphosphate (ATP—gamma, γ @ −2.5 ppm, alpha, α @ −7.5 ppm, and beta, β @ −16 ppm), phosphocreatine (PCr @ 0 ppm), and inorganic phosphate (Pi—intracellular (i) @ ~4.9 ppm and extracellular (e) @ ~5.2 ppm). Spectral peaks for phospho‐ mono‐ and di‐esters were observed. For tracking ATP flux rates multiple amplitude‐modulated (selective) RF pulses of constant maximum amplitude and length (2.06 μT, 114.29 ms) were applied on the γ‐ATP resonance. Chemical exchange of this phosphorus nuclei resulted in observable drops in signal for PCr (creatine kinase pathway) and Pi (ATP synthase pathway). N.B. control experiments applied 30*MT pulses at 15 ppm to account for saturation of the underlying phospholipids.

Following positioning of the head at the magnetic isocentre of the scanner, a ^1^H‐based *T*_2_ localiser was acquired (3 slice packages; 15 slices; 256 mm^2^ field of view (FOV); 6 mm slice thickness; repetition time (TR) 606 ms; echo time (TE) 122 ms; number of averages (NA) 1, flip angle (FA) 150°; 60% phase resolution; 6/8 phase partial Fourier; 592 Hz/px; echo spacing 4.06 ms; turbo factor 154; RF pulse type—fast; gradient mode—fast). This tri‐planar localiser was used to position a cuboid adjustment window covering the entire visual cortex (the size and position simply relating to the visible area of the individual brain due to the use of a surface coil—Figure 3a). Manual frequency, power, and 3D shimming adjustments were completed to achieve a ^1^H water peak with full width at half maximum (FWHM) of 19.9 ± 1.0 Hz and ^31^P PCr peak with FWHM of 16.5 ± 0.97. 3D shimming utilised a multiple gradient echo field mapping approach with resolution optimised for brain imaging. The PCr resonance was set to 0 ppm. This was confirmed by a short ^31^P FID‐based acquisition (TR 4000 ms; NA 32; FA 90°; bandwidth (BW) 4000 Hz; acquisition duration 512 ms; spectral points 2048) further used to find the exact frequency offset for γ‐ATP.

A non‐localised ^31^P free induction decay‐based magnetisation/saturation transfer (MT) sequence with narrowband selective saturation was developed in‐house (Figure 2c) following Chen et al. (2018). The MT sequence pulse train was comprised of multiple amplitude‐modulated RF pulses (hyperbolic secant in shape) of constant maximum amplitude and length (2.06 μT, 114.29 ms) interleaved with spoiler gradients (5 ms, 10 mT/m) in G_x_, G_y,_ and G_z_ and a 50 ms delay time. Hyperbolic secant pulses are adiabatic and therefore less sensitive to variations in B1 intensity from the use of a surface coil (by several orders of magnitude). Following the MT pulse train, a standard pulse‐acquire excitation regime (nominal FA 90°) was applied. B1 field inhomogeneity here limits the signal to the cortex underlying the surface coil (as visualised in Figure 3a).

The MT RF pulse allowed sufficient irradiation of the ^31^P γ‐ATP resonance (@ approx. −2.5 ppm) but was selective enough to have negligible sidebands. To assess the selectivity of the MT pulse, ^31^P spectra were acquired across three experiments with 30 pulses applied off‐centre at (i) −2.5 ppm (the intended γ‐ATP resonance), (ii) +2.5 ppm (the chemical shift of phosphodiesters, PDE), and (iii) +15 ppm (off‐resonance). As expected (due to limited chemical exchange between the target species), no drop in signal for Pi (@ 5 ppm) or PCr (@ 0 ppm) was observed when the MT pulse was applied at +2.5 ppm, confirming suitable selectivity. Off‐resonance saturation with 30 pulses at +15 ppm was used as the baseline condition (0 s saturation time). Phospholipid molecular rotation is fast compared to the saturation time, resulting in a broad resonance under the peaks of interest (Kwee & Nakada, 1988; McNamara et al., 1994). RF saturation at −2.5 ppm will therefore affect both phospholipids and γ‐ATP. Saturation at 15 ppm effects only phospholipids, resulting in comparable baselines (Dekruijff et al., 1980).

The time permitted for chemical exchange between ATP and PCr or ATP and Pi (or saturation transfer time, t_sat_) was varied across seven separate scans by changing the number of RF pulses in the MT train. The number of pulses was varied as 0, 3, 6, 12, 18, 30, 44 × 169 ms (RF pulse 114 ms + delay 50 ms + spoiler gradients 5 ms), resulting in saturation transfer times (*τ*) of ~0, 0.5, 1.0, 2.0, 3.0, 5.0, and 7.4 s. The order of the seven scans was randomised for each participant at each visit. A pause of 60 s was applied between each MT scan to allow for complete spin relaxation to equilibrium. Other scan parameters were as follows: NA = 32; TR = 12,000 ms; BW = 3000 Hz; data points = 2048; acquisition duration 692 ms. The total scan time, including setup, was approximately 80 min.

#### 2.2.2 Spectral analysis

The ^31^P spectral data were analysed offline in MATLAB 2020a (The MathWorks, Natick) using software routines developed in‐house. Raw frequency data underwent 5 Hz line broadening (Gaussian filter) to improve SNR. Spectra were manually phased (0th and 1st order correction). Following the Fourier transform, the baseline was fitted to a fourth‐order polynomial and removed. Resultant spectral peaks were assigned to the following ^31^P resonances: phosphomonoesters (PME), Pi (intracellular ~4.9 ppm and extracellular ~5.3 ppm), phosphodiesters (PDE), PCr, and the three resonances of ATP (γ‐ATP, α‐ATP, and β‐ATP) (Figure 3b).

To quantify signal contributions from intracellular (Pi), the spectra were further windowed between 4 and 6 ppm. Data were fitted, using nonlinear least squares with a Levenberg–Marquardt algorithm, to appropriate Gaussian/Lorentzian functions for Pi(i) and Pi(e) with chemical shift, amplitude, and FWHM floating variables. The baseline (raised due to phospholipid contributions) was again fitted to a fourth‐order polynomial. Fitting parameters were used to isolate the signal contribution from intracellular Pi. The area under the curve was extracted as a function of saturation time for the seven scans. All data were normalised to the control (15 ppm offset) experiment.

The formation of ATP via the ATP synthase pathway (aerobic) and the creatine kinase pathway (anaerobic) are in equilibrium (Figure 1). Therefore, signal saturation of the terminal phosphate (γ) of ATP, with a pulse train of RF, induces a signal decrease in both PCr (due to the CK equilibrium) and P_i_ (due to oxidative phosphorylation). The signal reduction, *S*(*τ*), for either Pi/PCr, as a function of saturation time, *τ*, follows (Forsen & Hoffman, 1963):

(1) $$S\left(\tau \right)=S\left(0\right)\left\{\left(k_{f}.T_{\mathrm{app}}\right)e^{-\left(\tau /T_{\mathrm{app}}\right)}+\frac{T_{\mathrm{app}}}{T_{1}}\right\}$$

where, *k*_*f*_ is the forward ATP exchange rate, *T*_1_ is the intrinsic relaxation time of PCr/Pi, and the “apparent” decay time constant, *T*_app_, is:

(2) $$\frac{1}{T_{\mathrm{app}}}=\frac{1}{T_{1}}+k_{f}$$

Note the longitudinal relaxation time, *T*_1_, of Pi and PCr are longer than the lifetime of ATP (Befroy et al., 2012; Liu et al., 2017) at clinically relevant MR field strengths. By varying saturation time (achieved by changing the number of pulses in the pulse train—Figure 2c) and measuring signal levels (integrals), one can therefore estimate the forward exchange rate of ATP for both pathways by fitting resultant MT ^31^P‐MRS data to equations 1 and 2 (Bittl & Ingwall, 1985). We followed Chen et al. and used a fixed 3.0 s for the *T*_1_ of Pi(i), to extract the rate of ATP flux, *k*_*f*_ (analysis was repeated for PCr with an assumed *T*_1_ of 5.1 s) (Chen et al., 2018).

^31^P MRS data was used to extract intracellular pH. Using equation 3 (Cichocka et al., 2015), we estimated pH pre‐ and post‐PBM.

(3) $$\mathrm{pH}=6.66+\log_{10}\left(\frac{\delta_{\mathrm{pi}}-3.08}{5.57-\delta_{\mathrm{pi}}}\right)$$

#### 2.2.3 Statistics

Due to the relatively small sample size and non‐normally distributed data (Kolmogorov–Smirnov test of normality, *D* = 0.262, *p* = 0.010), non‐parametric statistics (Wilcoxon Signed Ranks test, two‐tailed) were applied to compare pre‐ and post‐PBM *k*_*f*_ rates for both metabolic pathways (Pi and PCr). As participants varied in age (60–85 years), a Kendall's tau (non‐parametric) correlation was performed between age (years) and the difference in k‐rate before and after PBM treatment to assess whether age played a significant role in PBM effects.

## 3 RESULTS

### 3.1 General observations

Over the 5‐day treatment period, none of the 10 participants reported uncomfortable/adverse heating of the scalp due to PBM application over the 20‐min application time. This is in agreement with theoretical data from Ibrahimi and Delrobaei (2022) showing that changes in the tissue's temperature are minor. The ^31^P MT pulse sequence developed remained within safe Specific Absorption Rate (SAR) constraints for MRI, and no participant reported feeling hot or uncomfortable during the 50‐min ^31^P MT‐MRS measurement time.

Of the 10 participants who completed the study, three were excluded from analysis. One participant self‐reported low‐level migraine each morning that were present on Day 1 of the study (baseline MRS data collection) but had dissipated by Day 5 (follow‐up MRS data collection). We excluded this participant because the pathophysiology of migraine in terms of vascular contributions is still debated (Mason & Russo, 2018). Changes in cerebral blood flow and potential cortical spreading depression events (Charles & Brennan, 2009) associated with migraine (Feuerstein et al., 2016; Grech et al., 2021) can drive energetic imbalance. A second participant had an involuntary movement condition. This resulted in increased noise in the spectral measurements, which made it difficult to identify a suitable Pi peak. A third participant required a comfort break during the baseline (Day 1) MRS measurement and had to be removed from the scanner. Two points on the MT decay curve were measured before the break and five after, confounding the analysis.

Due to on‐going COVID‐19 disruptions, two of the remaining seven participants could not return on Day 5 of the experiment. Treatment was extended to 7 days, and these two participants returned for ^31^P MRS assessment on Day 8. This is noted in the subsequent analysis.

General observations regarding specific age, skin tone, and hair colour/coverage for each participant are detailed in Table S1. Skin tone and hair colour/coverage being important as the pigment melanin will scatter red light more, and therefore, depending on the colour and thickness, could present a significant barrier to light penetration depth.

### 3.2 ^31^P magnetisation transfer spectroscopy

Representative ^31^P spectra from the human visual cortex are shown in Figure 3. Key metabolites were easily identifiable. When applying the MT pulse train (30 pulses) off‐resonance at 15 ppm (control spectrum), we observed clear peaks for the three phosphorus sites of ATP (ATP—gamma, γ @ −2.5 ppm, alpha, α @ −7.5 ppm, and beta, β @ −16 ppm, PCr @ 0 ppm, and Pi—intracellular (i) @ ~4.9 ppm, and extracellular (e) @ ~5.2 ppm). Spectral peaks for phospho mono‐ and di‐esters were also observed.

With the MT pulse train (44 pulses—longest saturation time of 7.4 s) off‐resonance at −2.5 ppm (chemical shift of γ‐ATP), we see comparable spectra to those acquired with saturation at +15 ppm. This is direct evidence that a similar suppression of the phospholipid baseline was achieved between the conditions. While the off‐resonance (control) 5 s saturation time (30 pulses in the MT train) provides adequate phospholipid suppression, we did not explore if this could be achieved with smaller saturation times.

Importantly, in the ^31^P MT spectra we see a clear/effective saturation of the target γ‐ATP peak. Figure 3b shows the relative difference between this spectrum and the above “control” (off‐resonance) condition. We also observe drops in peak amplitude for Pi and PCr due to chemical exchange as part of aerobic and anaerobic metabolic process outlined in the introduction. We note that while the associated drop in Pi signal amplitude is small, it is easily detectable by eye at the long saturation times used in this experiment (see Figure 3b insert).

The chemical shift difference between PCr and γ‐ATP is only ∼2.5 ppm, and partial suppression of PCr while saturating γ‐ATP has been documented (Jeong et al., 2011). Hence, in an additional control experiment, we moved the centre frequency of the saturation pulses to 2.5 ppm to quantify any partial suppression effects. By following Chen et al. (2018) and using adiabatic hyperbolic secant pulses, we see negligible direct saturation due to RF bleed (rather than chemical exchange) when using these highly selective pulses (data not shown). No further corrections to the data were applied.

**FIGURE 4:**
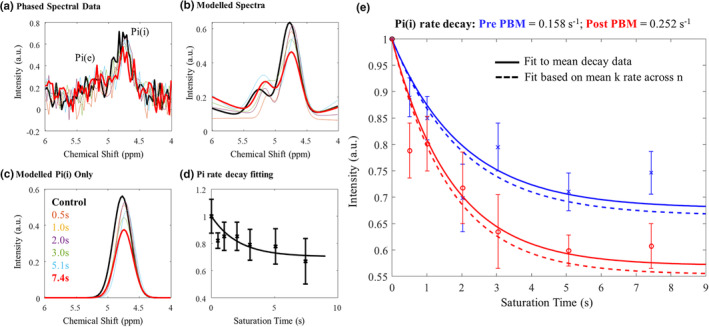
Data analysis pipeline for MT‐^31^P MRS data. (a) Raw data is filtered (exponential windowing function) and phased before Fourier transform and baseline correction (4th order polynomial). Data were windowed between 4 and 6 ppm to isolate Pi peaks; (b) Resultant spectra were fitted to appropriate Lorentzian functions for Pi(i) & Pi(e) (chemical shift, amplitude, and FWHM free variables); (c) Signal contributions from Pi(i) were isolated for each saturation duration and normalised (to no saturation control) area under the curve taken; (d) data were plotted against saturation time and the decay fitted to estimate ATP flux rate (equation 1). Note plots a–d are representative data from one participant. (e) ATP flux rate estimates pre‐(blue) and post‐(red) PBM treatment based on signal decay as a function of RF saturation time across all participants. We find an increase in mean k‐rate for ATP production post‐PBM treatment. T_1_ of Pi(i) was fixed to a constant (3.1 s) pre‐ and post‐treatment. Curves were fitted for each individual participant and also for the mean decay data across participants. Standard errors across *n* = 7 participants shown.

Representative results for progressive saturation of γ‐ATP are shown in Figure 4a–d. Associated signal decreases in Pi(i), due to reversible chemical exchange as part of ATP synthase, were visible in the “raw” filtered spectral data at long saturation times (7.4 s—highlighted in Figure 4a). However, the inherent low SNR for this important metabolite (driven by the clinical field strength used (3 T) and the short acquisition period—6.4 min) meant accurate data quantification of peak area required data fitting (see methods). ^31^P MT‐based spectra (windowed between 4 and 6 ppm) were fitted to two Lorentzian functions corresponding to Pi(i) and Pi(e). Chemical shift, peak amplitude, and peak FWHM were floating variables. The resulting fits are shown in Figure 4b. Again, the drop in Pi(i) signal with increased saturation time is maintained and clearly observable. No similar trend was observed for Pi(e). This could reflect the lack of chemical exchange pathways between γ‐ATP and Pi(e) or the even lower SNR due to low tissue concentration. It is noted that there was still a significant baseline offset—even following phospholipid saturation. Therefore, a fourth‐order polynomial was fitted to the data, allowing the extraction of the peaks of interest and isolation, in this case, of the intracellular Pi fit (Figure 4c). The area under the curve was extracted and plotted as a function of saturation time (Figure 4d). For each saturation time, the error was estimated as the RMS error from the fitting residual. An exponential decrease in signal was observed as a function of saturation time. This was fitted to equation 1 to estimate the ATP flux rate (*k*_*f*_) across all participants (Figure 4e).

**FIGURE 5:**
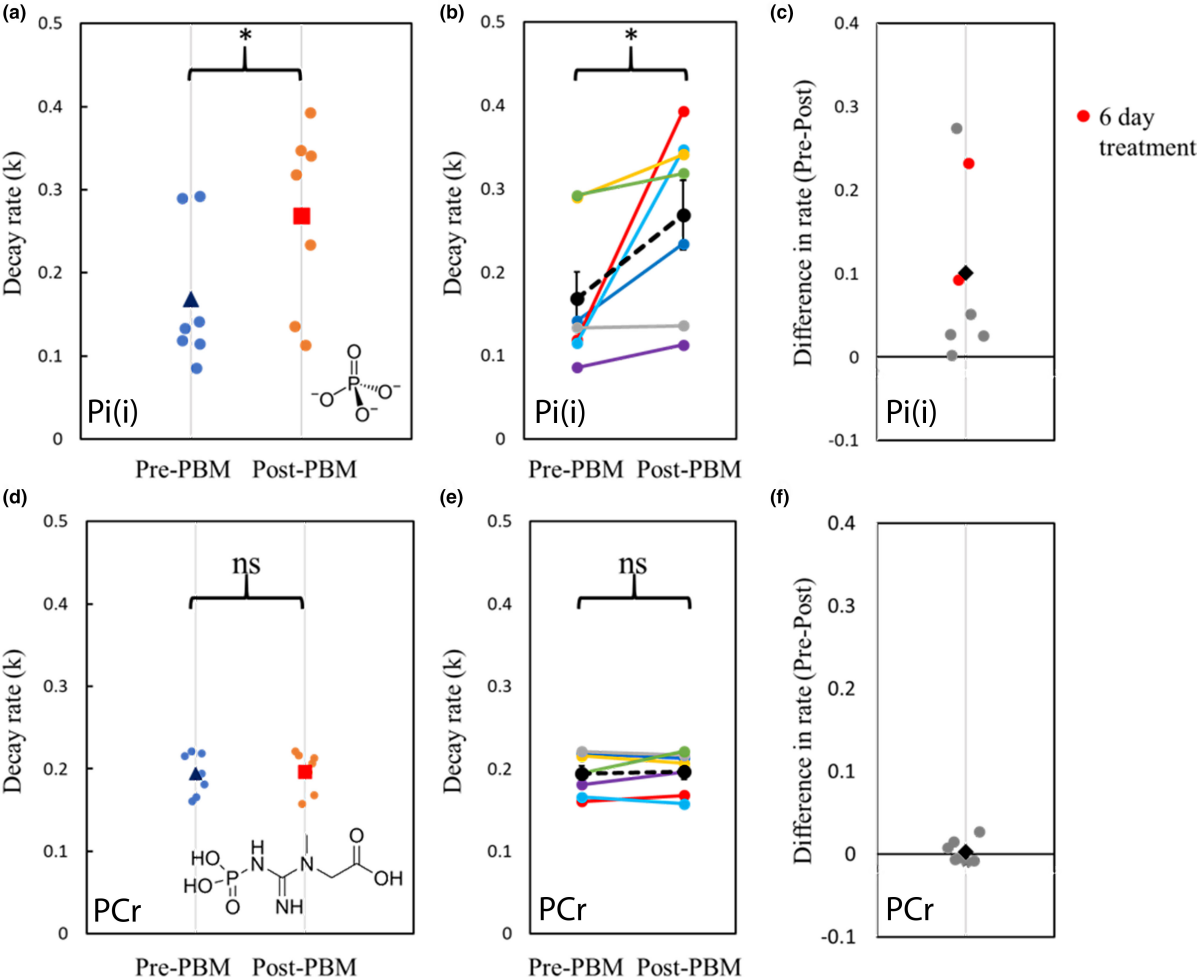
Exploration of key rates of decay before and after PBM treatment. (a) Relative decay rate (k) of inorganic phosphate pre‐ & post‐PBM treatment. Small dots represent individual data. Blue triangle: mean k‐rate pre‐PBM. Orange square: mean k‐rate post‐PBM; (b) Relative decay rate (k) of inorganic phosphate pre‐ and post‐PBM. Coloured lines indicate individual data pairs (*n* = 7). Dashed black line and dots represent the mean of all participants; (c) Difference in k‐rate (post‐PBM–pre‐PBM). Small dots indicate individual data. Black diamond represents the mean difference across participants. Participants who had extended PBM application time (7 days) are highlighted in red; (d) Relative decay rate (k) of phosphocreatine pre‐ and post‐PBM treatment. Small dots represent individual data. Blue triangle: mean k‐rate pre‐PBM. Orange square: mean k‐rate post‐PBM; (e) Relative decay rate (k) of phosphocreatine pre‐ and post‐PBM. Coloured lines indicate individual data. Dashed black line and dots represent the mean of all participants; (f) Difference in k‐rate (post‐PBM–pre‐PBM). Small dots indicate individual data. Black diamond represents the mean difference across participants.

Note that while these data can be used to fit both forward ATP flux (*k*_*f*_) and *T*_1_ for Pi(i), with only seven data points in the extracted exponential decay and relatively high noise for each point, floating both variables caused the fitting algorithm to hit the boundary conditions for *T*_1_(Pi(i)) in all cases (1 or 7 s). We therefore followed Chen et al. (2018) and fixed *T*_1_ at 3.1 s and only extracted *k*_*f*_ for each individual participant (*n* = 7) and for the mean decay data (see Figure 5c).

Fitting to the mean decay curve across *n* = 7 participants pre‐ and post‐PBM application, we found a *k*_*f*_ rate for ATP synthase of 0.158 and 0.252 s^−1^, respectively. Fitting each individual decay curve and then averaging extracted *k*_*f*_ rates, we found 0.198 ± 0.035 s^−1^ and 0.298 ± 0.045 s^−1^ (standard error) pre‐ and post‐PBM, respectively (see Appendix S1 for individual fitting data). We found an increase in *k*_*f*_ rate for ATP production post‐PBM compared to pre‐PBM application. For transparency, when data from the three excluded participants (see above) are included, the extracted *k*_*f*_ becomes 0.182 ± 0.025 s^−1^ and 0.208 ± 0.044 s^−1^ pre‐ and post‐PBM, respectively.

Figures 5a–c summarise the changes in the rate of decay of Pi before and after PBM treatment. The rate increased in six of the seven participants and remained stable in one. Because of the small sample size and non‐normally distributed data (Kolmogorov–Smirnov test of normality, *D* = 0.262, *p* = 0.010), non‐parametric statistics were applied to compare pre‐ and post‐PBM rates. The decay rate for Pi (intracellular) was significantly faster post‐PBM compared to pre‐PBM (Wilcoxon signed rank test, two‐tailed, *Z* = −2.366, *p* = 0.016).

To determine if participant age influenced the change in k‐rate of Pi, a Kendall's tau (non‐parametric) correlation was performed between age (years) and the difference in k‐rate pre‐ and post‐PBM treatment. The relationship between age and Pi k‐rate change was not significant (Kendall's tau *b* = −0.293, *p* = 0.362), indicating that age was not a significant confound within the range tested (60–85 years).

To verify that the increase in rate of ATP synthase post‐PBM treatment did not simply reflect a whole‐body change in metabolism, we applied the same analysis (with *T*_1_ set to 5.1 s) to explore creatine kinase‐based anaerobic metabolic pathways. Figures 5d–f summarises the changes in the rate of decay of PCr before and after PBM treatment. In contrast to Pi, there was no significant change in decay rates of PCr before and after PBM treatment (Wilcoxon signed rank test, two‐tailed, *Z* = −0.338, *p* = 0.813).

It is noted that participants undergoing either 4‐ or 7‐day PBM treatment showed significant increases in Pi(i) to ATP flux rate. However, there was no apparent association between the length of PBM treatment and *k*_*f*_ rate change across our limited cohort (Figure 5c).

We estimated pH pre‐ and post‐PBM (Cichocka et al., 2015). While there is a small increase in pH post‐PBM (7.54 ± 0.01 compared to 7.50 ± 0.01 pre‐PBM) this is within standard error and therefore likely not a driver or reflective of the measured increase in ATP synthase flux.

## 4 DISCUSSION

It has been previously hypothesised across the literature that light activation of the ETC mediator cytochrome c and the associated protein CCO drive increases in ATP synthase exchange rate. Using MT ^31^P MRS, we have quantified a significant increase in neuronal aerobic ATP flux in response to 670 nm PBM treatment over 5 days in a small cohort of aged (60 years+) healthy participants. There is strong evidence for direct glycolysis/mitochondrial involvement, as we found no observable increase via the creatine kinase pathway. Interestingly, we found no statistical significance (Kendall's tau *b* = −0.293, *p* = 0.362) between k‐rate change (pre‐ to post‐PBM) and age across our cohort. This could be reflective of (i) the small sample size or (ii) differences in pre‐PBM (baseline) k‐rates, which one could assume relate to biomarkers of mitochondrial dysfunction (i.e., lower initial rates represent impaired cognitive function in age). It is therefore imperative that future studies incorporate cognitive testing to the experimental design to assess this. Additional testing post‐PBM treatment would demonstrate if indeed there is any cognitive improvement related to the observed increase in ATP synthase rate here. Unfortunately, project data here was collected shortly after the first UK COVID‐19 lockdown, and laboratory access for wider cognitive testing was limited. Complementary functional near‐infrared spectroscopy (fNIRs) studies, illuminating the forehead/prefrontal cortices with sources at 840 and 770 nm, do demonstrate faster reaction times and better accuracy in cognitive tests (Waight et al., 2023), but do not quantify changes in metabolism directly as we do here.

It is noted that the pigment melanin in the skin & hair, along with the fibrous/cylindrical structure of the hair, could also play an important role in attenuating light flux to the cortex and, hence, influencing the magnitude of the change in the k‐rate of Pi. Melanin has a photoprotective action because of its light absorption properties (Kollias et al., 1991). It strongly absorbs light in the ultraviolet (UV) and visible ranges, but importantly, it also has low absorption in the near‐infrared range (Zonios et al., 2001). Melanin will also scatter more red light for increased refraction. Therefore, darker hair/skin tones (increased melanin) will reduce the photon flux to the cortex. With age, we lose melanin, and our hair can turn grey/white. Hence, with age, one could assume that only hair thickness will determine cortical light flux (with thicker hair scattering/refracting more photons away from the scalp). Observation‐based data (detailed in Table S1) highlights the limitation of the current study in that we used a small cohort of participants with a light skin tone (all white British in ethnicity). In addition, due to the age range tested, most of the cohort had grey hair. While our one participant with thick brown hair (in a bob) did show only a small increase (k = 0.292 [pre] ➔ 0.318 [post]) in k‐rate pre‐ to post‐PBM, suggesting that hair colour does represent a treatment barrier, the smallest increase (k = 0.133 [pre] ➔ 0.136 [post]) was counterintuitively observed in a participant with short grey hair. Across the current cohort, the largest changes in k‐rate were observed in people with both thick white and shaved white hair. Again, this could be reflective of differences in pre‐PBM (baseline) k‐rate, or it could simply reflect PBM application compliance issues (in this single participant with grey hair). In short, while individual inferences are interesting, a wider study with much larger cohort numbers should be completed to explore the correlation of k‐rate to such general observations (incorporating more diversity in terms of skin tone and hair colour and actual qualitative measures taken, for example, the self‐reported 9‐point Self‐Assessment Skin Tone Palette test) (Martin et al., 2023; Nakashima et al., 2022). A larger cohort study should also include a control group with (i) a sham treatment (no emitted light) and (ii) a young group (18–30 years) where mitochondrial dysfunction should not be observed.

**FIGURE 6:**
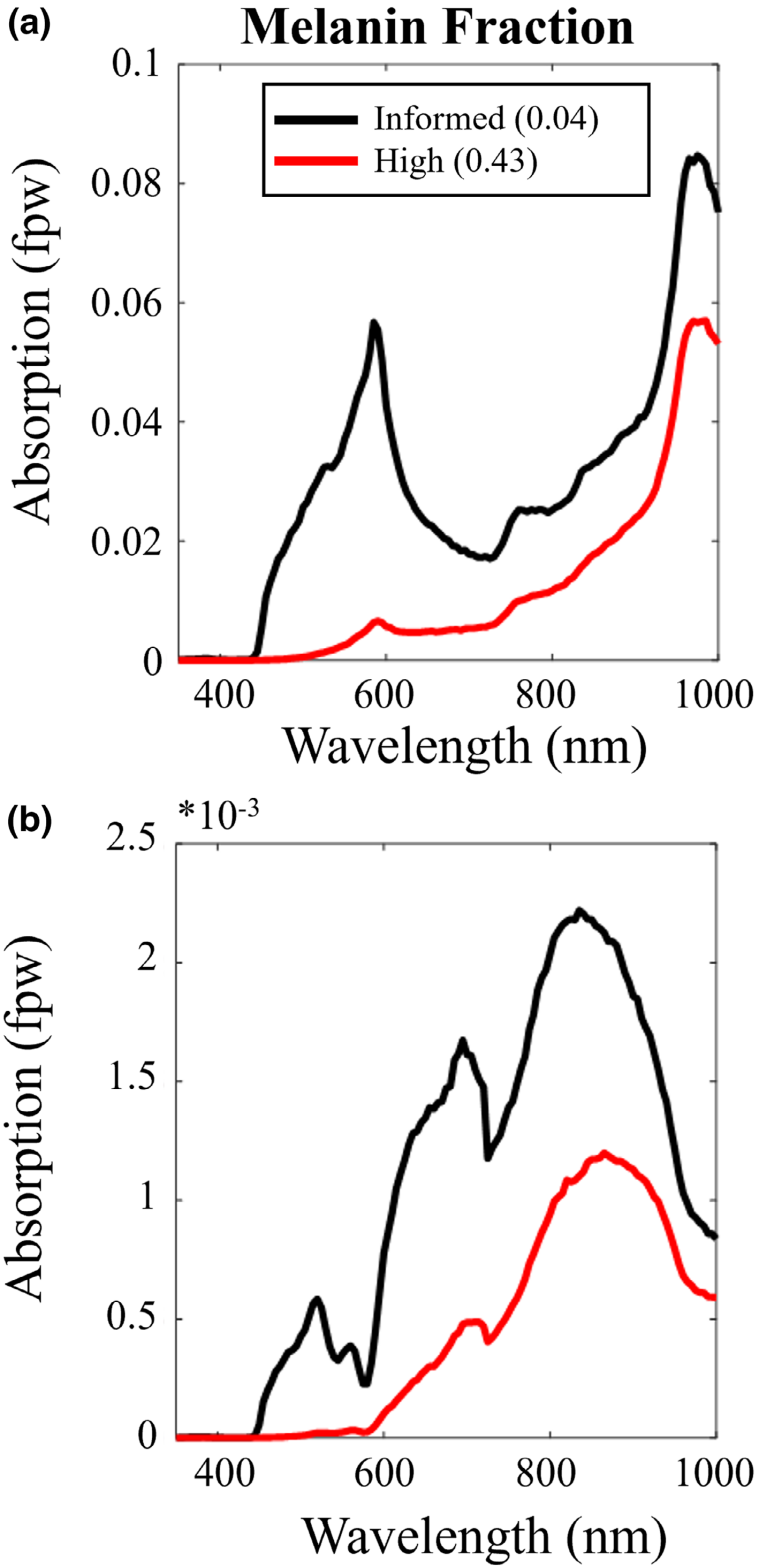
Simple layered Monte Carlo simulations allow exploration of absorbed light in (a) the cortical grey matter and (b) cytochrome c oxidase (via simple multiplication of the former by the fractional μ_A_ of the target chromophore. Peaks in photon absorption by cytochrome c ~670 nm and 810–830 nm. Melanin fraction has a marked effect on absorption.

Bias based on hair colour and skin tone in other optical‐based techniques (including pulse oximetry and fNIRs) relying on blood absorption‐based measures has recently come into focus (Dyer, 2020; Tobin & Jubran, 2022; Webb et al., 2022). Indeed, accurate modelling of light transport through tissue can help account for melanin effects. We, alongside others across the literature (Salehpour et al., 2019), complete layered 3D Monte Carlo simulations, following the original work of Laloy et al. (2013) and validated against Wang et al. (1995). Such simulations indicate that at any given wavelength (above 450 nm), less than 8% of the light transmitted into the head reaches the GM (Figure 6a). At the target wavelength (670 nm), around 2% of light is absorbed in GM. This basic simulation provided theoretical evidence that only ~0.16% of 670 nm photons incident on the GM are absorbed by CCO (Figure 6b). Simulation data can be used to explore changing the melanin fraction in the skin layer from 0.04 (representing pale skin tones) to 0.43 (representing dark skin tones) (Hani et al., 2014) on the amount of light being absorbed by GM or cytochrome c in the brain (Figure 6). We demonstrate a dramatic 2/3 drop in the amount of 670 nm photons being absorbed by cytochrome c in GM under skin with a high melanin fraction. To mitigate this, either the incident light will have to be increased or the PBM light exposure time will have to be increased to overcome this loss. Increasing the energy density of the incident light while overcoming this potential PBM shortfall may impact physiology in other unknown ways. Note that, if the illumination spot size is large enough, the energy density required for effective PBM is believed to be low, in the range of 1–16 Joules/cm^2^. It has been shown that above a certain threshold (outside this dose window), increasing the energy delivered beyond ~3000 Joules in a session will not increase the therapeutic effects (Huang et al., 2011). Our study here delivered the recommended 20‐min treatment time over the course of several days to reach the optimum dose (~1900 Joules per session—see Table S4). While increasing exposure time here would help reach the upper bound on energy delivered, increasing energy/power density to overcome higher melanin absorption may become counterproductive and/or result in higher scalp temperatures (Ibrahimi & Delrobaei, 2022). Future experiments should incorporate more accurate tissue models of light transport to inform experimental design in terms of illumination duration/intensity. These layered models could be personalised based on high‐resolution MRI data to inform the thickness of each tissue layer (including skull and CSF, which can affect light scattering and heat transport (Beauchamp et al., 2011; Jiang et al., 2020)) which can affect the depth at which light penetrates the head (Cui et al., 2011; Haeussinger et al., 2011; Strangman et al., 2014). As computing power improves, models should also incorporate bio‐heat transfer modelling (Ibrahimi & Delrobaei, 2022) and account properly for light scattering from human hair fibres (Marschner et al., 2003).

Notwithstanding, we do observe a significant increase in ATP synthase rate post‐PBM treatment, and while such data may lend support to the theory that PBM activates CCO in the ETC, we acknowledge that our simple MCS data (Figure 6) shows that the number of photons reaching the mitochondria for absorption is extremely small (~0.15%). Importantly, this spectral simulation does indeed confirm that 670 nm represents a small peak wavelength for cytochrome c absorption in transcranial PBM, corresponding to absorption by the haem iron‐Met80 bond (Huettemann et al., 2011). Our data here thus confirm the Wong‐Riley et al. (2005) study, which illuminated primary neurons (with 670, 728, 770, 830, and 880 nm light) functionally inactivated by toxins (Wong‐Riley et al., 2005). The greatest increase in energy metabolism occurred under 830 and 670 nm light. The least effective wavelength was found to be 728 nm, which indicates this wavelength will be less effective for PBM therapy if cytochrome c absorption is the hypothesised mechanism—finding potential use as a sham control in future studies. The later Wong‐Riley et al. (2005) study detailed that LED treatment at 670 nm significantly reversed the detrimental effect of a toxin on neurons using a similar cumulative dosage (*p* < 0.001 for three metabolic neuronal types) (Wong‐Riley et al., 2005). However, simulations here (and by others across the literature) show that if cytochrome absorption is the driving mechanism, applying PBM at 810–820 nm (corresponding to the copper centres) would improve therapeutic efficacy. Peak cytochrome absorption occurs between 810 and 820 nm at 0.2% (Figures 1c–e and 6b). Furthermore, the drop in photon absorption is less pronounced (1/2 photon count) at this optimum 820 nm peak, reducing the bias due to skin tone. This finding is in line with Jagdeo et al. (2012), who demonstrated using a cadaver skull with intact soft tissue that near‐infrared light at 830 nm penetrated further than red light at 633 nm (Temporal region: 0.9% @ 830 nm, 0% @ 633 nm; frontal region: 2.1% @ 830 nm, 0.5% @ 633 nm; occipital region: 11.7% @ 830 nm, 0.7% @ 633 nm) (Jagdeo et al., 2012). However, from a practical self‐application perspective, the visible nature of the 670 nm light from this bulb in the present study we believe helped with treatment compliance (our aged participants could tell the bulb was on as it was red). Compliance may become an issue at 820 nm (a wavelength invisible to the naked eye). Again, based on the present data, future studies should explore this.

Alternative mechanisms to cytochrome c absorption explaining the improved mitochondrial function with PBM are widely discussed across the literature (Hamblin, 2016; Sommer, 2019). Moreover, other physiological confounds could be driving the increase in ATP flux rate measured here. Recent studies have demonstrated that PBM directly affects cerebral blood flow, CBF, cerebral oxygenation (Tian et al., 2016), regulation (via nitric oxide generation) (Baik et al., 2021; Chao, 2019; Hamblin, 2018; Salgado et al., 2015), although such effects have been shown to be somewhat wavelength dependent (Iennaco, 2001). Nevertheless, it is known that with age tissue pO_2_ drops (Moeini et al., 2018). Therefore, one could hypothesise that an increase in CBF could drive increases in oxygen extraction fraction under such modulated oxygen concentration gradients (between blood vessels and tissue). This would positively affect cognitive function, as others have found (Baik et al., 2021). By what amplitude and for how long CBF remains elevated after PBM treatment could be determined as part of future experiments utilising quantified arterial spin labelling (ASL) approaches (Buxton, 2005). The positive effects of PBM treatment have been found to last days, weeks, and months after treatment (Hamblin, 2016; Mitrofanis, 2017; Rojas & Gonzalez‐Lima, 2011). If CBF elevation is a key driver, it too should remain elevated and could be assessed as part of a longitudinal MRI‐based study. Such measures can in addition inform bio‐heat transfer models where it is assumed that vasodilation occurs in part to cool the cranium, specifically scalp and cortex. Temperature changes can be limited to 0.5 and 0.03°C, respectively (safe physiologically), during a 20‐min illumination duration (Ibrahimi & Delrobaei, 2022). To date, such models do not explore vasodilation after illumination. It is often assumed that blood flow will return to basal conditions.

One could assume that no matter the mechanism, strategies to increase the number of incident photons would result in further increases in ATP flux. Other alternatives to transcranial light delivery to overcome the barrier of the scalp and skull include intracranial and intranasal (Saltmarche et al., 2017) for delivery of the light directly through the oral cavity or through the retina; however, all of these are more or less invasive to varying degrees and are dependent on blood absorption and which area of the brain the therapy is needed.

The present study details a robust experimental protocol using ^31^P MRS with MT to quantify changes in ATP flux in response to longitudinal PBM through the identification of key metabolites. Although MR is less sensitive to ^31^P (in comparison to ^1^H), it does have a broad chemical shift range, which means fewer overlapping peaks and less complicated spectra. We do, however, note that the small signal amplitude of Pi can limit the quantification of ATP synthesis with MT‐based ^31^P MRS. Preclinical studies with progressive saturation transfer at high magnetic fields (11.7 T) easily differentiate/resolve the intra‐ and extracellular Pi pools (Tiret et al., 2016) noted that failing to accurately resolve extracellular Pi can cause a significant bias in the estimation of the forward constant rate of ATP synthesis. However, in the present study, we demonstrate that it is still possible to extract important metabolic parameters even at clinically relevant MRI field strengths (3 T) with suitable model fitting and data filtering.

While the exact mechanisms of magnetisation/saturation transfer effects are still debated (Balaban & Koretsky, 2011; Befroy et al., 2012; From & Ugurbil, 2011; Kemp & Brindle, 2012), one could alternatively use inversion transfer (IT) regimes. The main advantages of using IT to quantify exchange kinetics (Buehler et al., 2015; Degani et al., 1985) is that (i) it does not require long saturation pulses; (ii) It is less susceptible to unintended MT effects from small pools of metabolites; and (iii) the sensitivity to ATP synthesis rate can be enhanced by inverting PCr and all ATP resonances (Ren, Sherry, et al., 2015). In this way, the recovery of γ‐ATP is significantly delayed, which results in amplified MT effects between γ‐ATP and Pi. The disadvantage is that more comprehensive modelling is required to fully account for multiple magnetisation exchanges, including the cross‐relaxation between γ‐ATP and β‐ATP (i.e., the nuclear Overhauser effect (Ren, Yang, et al., 2015)). This method should be tested in response to PBM treatment but would require investment in RF coils capable of transmitting a homogenous 180° B_1_ field and consideration of SAR.

Chaumeil et al. (2009) performed a multimodal imaging study based on the combination of three neuroimaging techniques using ^18^F‐FDG PET (glucose consumption), indirect ^13^C MRS (TCA cycle flux) and ^31^P MT‐MRS (rate of ATP synthesis). The consistency of these three techniques for measuring metabolic fluxes does demonstrate the robustness of MT ^31^P MRS for directly evaluating ATP synthesis in the living brain across species (Chaumeil et al., 2009; Du et al., 2008; Lei et al., 2003). The ^31^P MT‐MRS method (with variable saturation time) deployed here to validate the metabolic benefits of PBM could be tested further by investigating exercise related increases in creatine kinase‐based metabolism. Following exercise, one should expect to observe a change in ATP k‐rate based on the PCr peak changes, without a change in the Pi peak. Future experiments could also compare the change in Pi‐related ATP production with PBM treatment in a young cohort, as PBM is purported to be most effective in older animals/humans (Shinhmar et al., 2020). Finally, research using similar methods could be extended to patients with neurodegenerative disease in which mitochondrial function (and hence ATP production) is further impaired. This would help validate that PBM is indeed a useful neuroprotective technique in ageing and disease. It has been reported through the NADPARK study (Brakedal et al., 2022) that nicotinamide riboside taken as an oral daily supplement improves mitochondrial function in early‐stage PD. Our ^31^P MT‐MRS method could be used to measure this improvement in terms of ATP synthesis rate and one could envisage using a combined treatment of transcranial PBM and nicotinamide riboside supplement. We are excited about the future of our refined PBM protocol for use in the fight against neurodegenerative disease for healthy ageing.

## AUTHOR CONTRIBUTIONS

Elizabeth J. Fear: Data curation, acquisition, and analysis. Validation. Project administration and writing. Frida H. Torkelsen: Data curation, acquisition, and Monte Carlo simulation. Project writing. Elisa Zamboni: Data analysis. Project writing, review, and editing. Kuan‐Ju Chen: Data analysis. Martin Scott: Data analysis. Writing review and editing. Glenn Jeffery: Visualisation. Writing review and editing. Heidi Baseler: Data curation and analysis. Project Management. Writing review and editing. Aneurin J. Kennerley: Data curation, acquisition, and analysis. Project Management. Writing review and editing.

## FUNDING INFORMATION

This project was funded by an EPSRC Impact Accelerator Award and Wellcome Trust Centre for Future Health award.

## CONFLICT OF INTEREST STATEMENT

None declared.

## Supporting information


Appendix S1

## Data Availability

The data that support the findings of this study are openly available in Mendeley at http://doi.org/10.17632/3r7kmfmpyj.1.
