# Supplementary material for: Use of 31P magnetisation transfer magnetic resonance spectroscopy to measure ATP changes after 670 nm transcranial photobiomodulation in older adults
Source: Aging Cell. 2023 Oct 6;22(11):e14005. doi: 10.1111/acel.14005 (PMC10652330; doi:10.1111/acel.14005)
Supplement: Supplementary file 1 — Appendix S1 [file ACEL-22-e14005-s001.docx]

# **Supplementary Information**

**Methods**

Table S1: Anonymised participant details including gender, age, number of days of light treatment, observations of hair colour and inclusion or removal from the study

| **Participant Number** | **Gender** | **Age** | **Ethnicity** | **Total Days of Light treatment** | **Hair colour** | **Removal /Inclusion** | **k rate (pre)** | **k rate (post)** |
| --- | --- | --- | --- | --- | --- | --- | --- | --- |
| 1 | M | 81 | White, British | 4 | very short grey hair | **Removed – Headaches ceased** |  |  |
| 2 | F | 70 | White, British | 6 | Blonde bobbed hair |  | 0.141539 | 0.234022 |
| 3 | F | 62 | White, British | 4 | long thick white hair |  | 0.118805 | 0.393007 |
| 4 | M | 67 | White, British | 6 | Very short shaved white hair |  | 0.115019 | 0.347228 |
| 5 | F | 60 | White, British | 4 | Thick grey, white hair |  | 0.289778 | 0.341039 |
| 6 | F | 66 | White, British | 4 | Short blonde bobbed hair | **Removed - a lot of movement** |  |  |
| 7 | F | 67 | White, British | 4 | short grey hair |  | 0.13358 | 0.135931 |
| 8 | M | 63 | White, British | 4 | short brown/grey hair | **Removed - due to toilet break** |  |  |
| 9 | F | 72 | White, British | 4 | brown bob |  | 0.292174 | 0.318338 |
| 10 | M | 73 | White, British | 4 | short grey hair |  | 0.085727 | 0.112768 |

Table S2: Device Information following standardised reporting for low level light therapy <https://doi.org/10.1089/pho.2011.9895>

| **Manufacturer** | **Model ID** | **Year Produced** | **Number of Emitters** | **Emitter Type** | **Emitter Spatial Distribution** | **Beam Delivery System** |
| --- | --- | --- | --- | --- | --- | --- |
| Red Light Man Ltd., Manchester, UK | Red Mini 670 | 2017 | 12 | LEDs | 12 emitters with a 30 degrees beam angle, covering a 78.54cm^2^ surface in a cross-like pattern | Delivery system mounted on a spring-loaded (angle-poise) adjustable arm |

Table S3: Irradiation Parameters following standardised reporting for low level light therapy <https://doi.org/10.1089/pho.2011.9895>

| ***Parameter*** | ***Value*** | ***Measurement method / information source*** |
| --- | --- | --- |
| **Centre wavelength [nm]** | 670 | Provided by manufacturer ([https://redlightman.com/product/red-mini-670/)](https://redlightman.com/product/red-mini-670/) |
| **Spectral bandwidth [nm]** | 620 – 700  (± 10) | Provided by manufacturer ([https://redlightman.com/product/red-mini-670/)](https://redlightman.com/product/red-mini-670/) |
| **Operating mode** | Continuous wave |  |
| **Frequency [Hz]** | 50/60 Hz | Provided by manufacturer ([https://redlightman.com/product/red-mini-670/)](https://redlightman.com/product/red-mini-670/) |
| **Pulse on duration [sec]** | NA |  |
| **Pulse off duration [sec]** | NA |  |
| **Peak radiant power [mW]** | 200 mW/cm^2^ at 0cm | Provided by manufacturer ([https://redlightman.com/product/red-mini-670/)](https://redlightman.com/product/red-mini-670/) |
| **Average radiant power [mW]** | 20 mW/cm^2^ at 60cm | Provided by manufacturer ([https://redlightman.com/product/red-mini-670/)](https://redlightman.com/product/red-mini-670/) |
| **Beam profile** | Gaussian | Provided by manufacturer ([https://redlightman.com/product/red-mini-670/)](https://redlightman.com/product/red-mini-670/) |

Table S4: Treatment Parameters following standardised reporting for low level light therapy <https://doi.org/10.1089/pho.2011.9895>

| ***Parameter*** | ***Value*** | ***Additional notes*** |
| --- | --- | --- |
| **Beam spot size at target [cm^2^]** | 87.25 cm2 | Given an initial diameter of 10cm, divergence (half-angle) of 15º, and distance of target of 1cm, the beam diameter is 10.54cm  Beam spot size at target is (10.54/2)^2*pi of 87.25 cm^2^ |
| **Irradiance at target [mW/cm^2^]** | 187 | Based on datapoints provided by manufacturer, irradiance at target (1cm from bulb) was derived at 187 mW/cm^2^ according to R2 goodness of fit for data interpolation |
| **Exposure duration [sec]** | 1200 | 20 minutes a day |
| **Radiant exposure [J/cm^2^]** | 0.0022 | Following H_e_ = E_v_ * t where E_v_ is the irradiance at target |
| **Radiant energy [J]** | 1900 | Radiant exposure (0.0022 J) * beam spot size at target (87.25 cm^2^) |
| **Number of points irradiated** | 1 | Region of the occipital lobe centered on the inion |
| **Area irradiated [cm^2^]** | 87.25 around the occipital lobe, centered on the inion |  |
| **Application technique** | The centre of the bulb was held 1 cm from the participant’s inion using a small spacer |  |
| **Number/frequency of treatment sessions** | 4 to 6 treatments total, delivered one every day over 4 to 6 days |  |
| **Total radiant energy [J]** | 7600 – 11400 | 1900 per day, ranging between 4 days and 6 days of treatment |

**Results:**

*Individual Fits per participant before and after photobiomodulation*


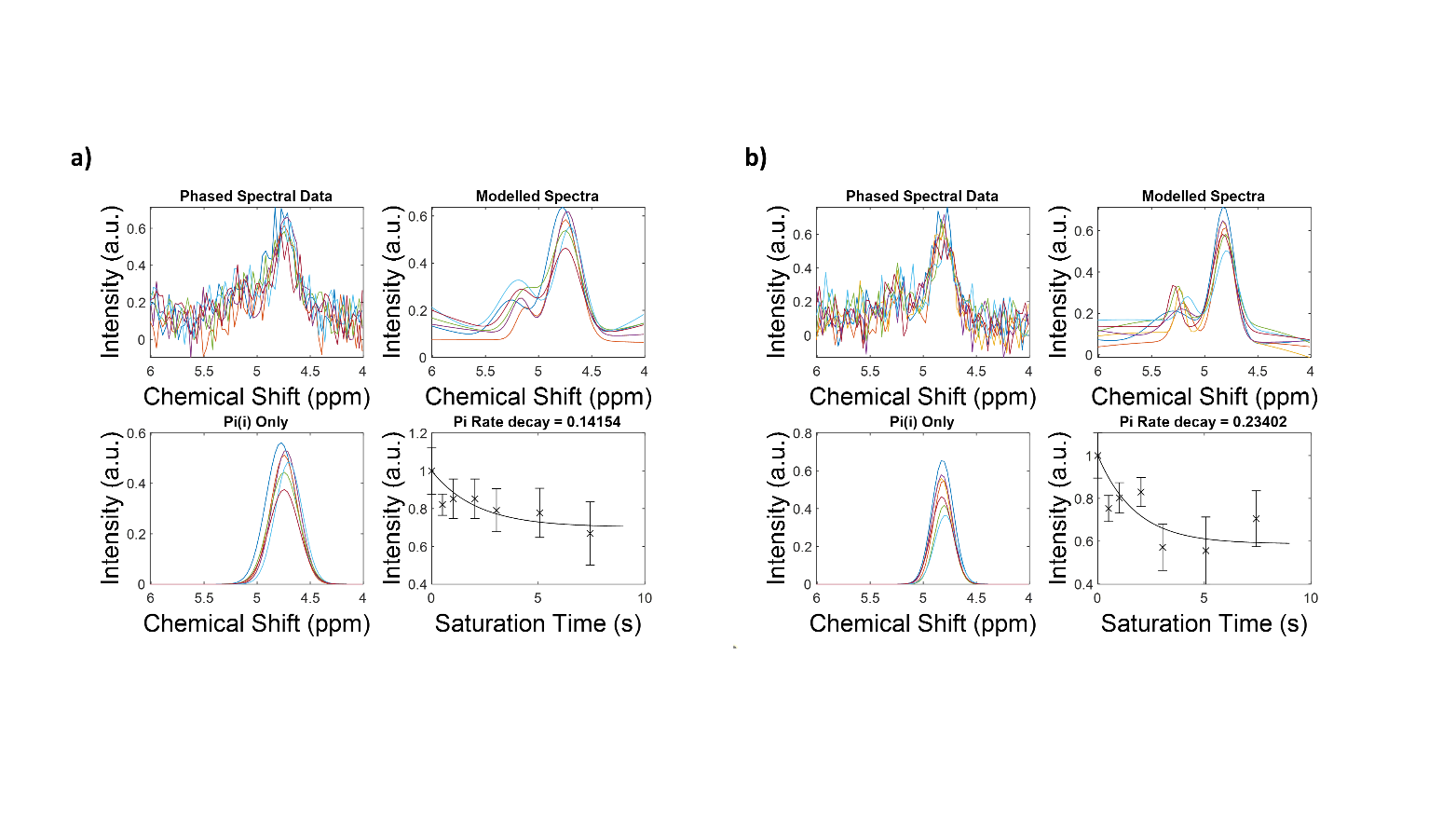


S1: ^31^P raw spectral data, processing of spectra and calculation of k rate for inorganic phosphate using the magnetisation transfer technique for Participant 2 a) pre b) post PBM treatment


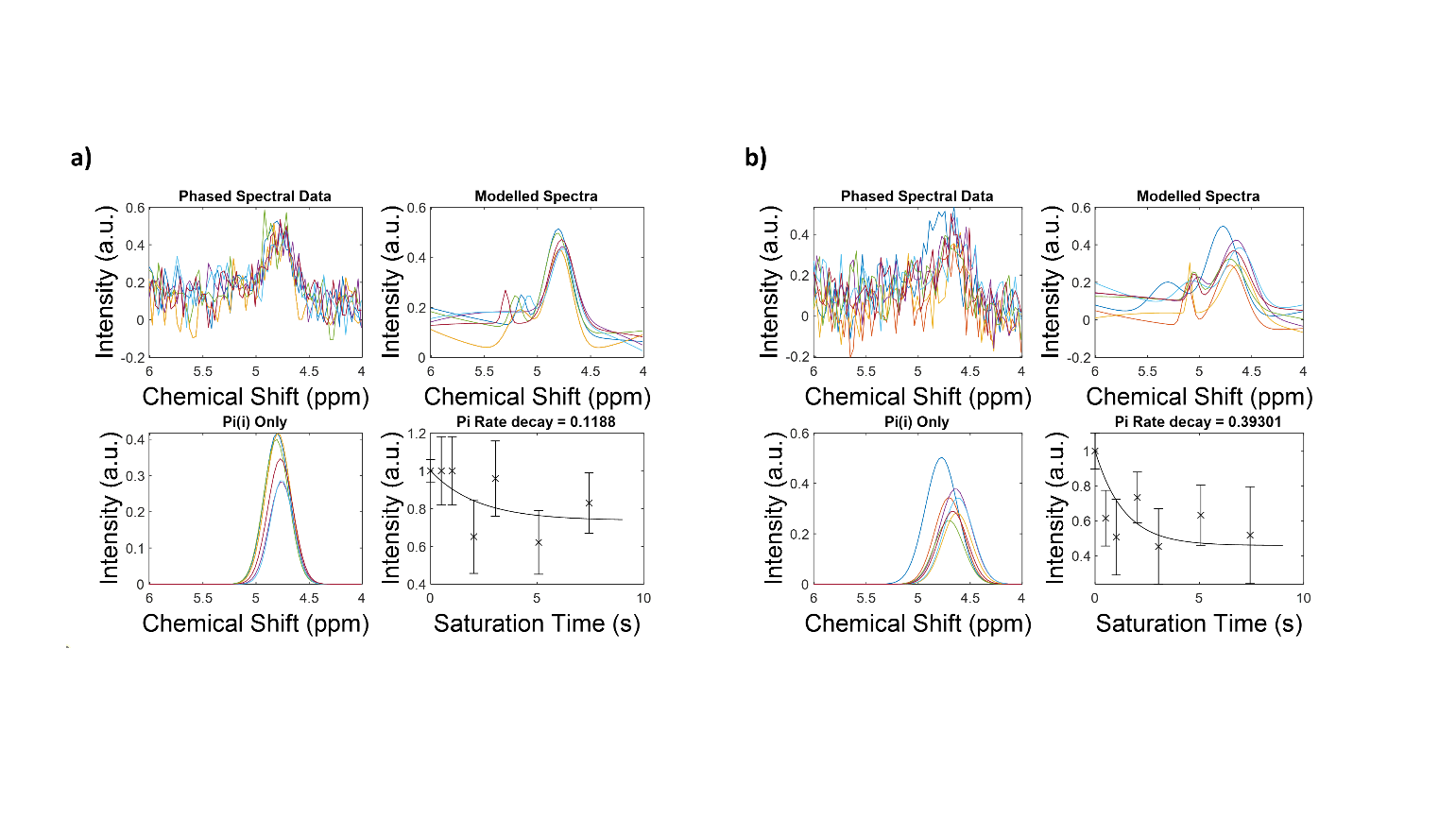


S2: ^31^P raw spectral data, processing of spectra and calculation of k rate for inorganic phosphate using the magnetisation transfer technique for Participant 3 a) pre b) post PBM treatment


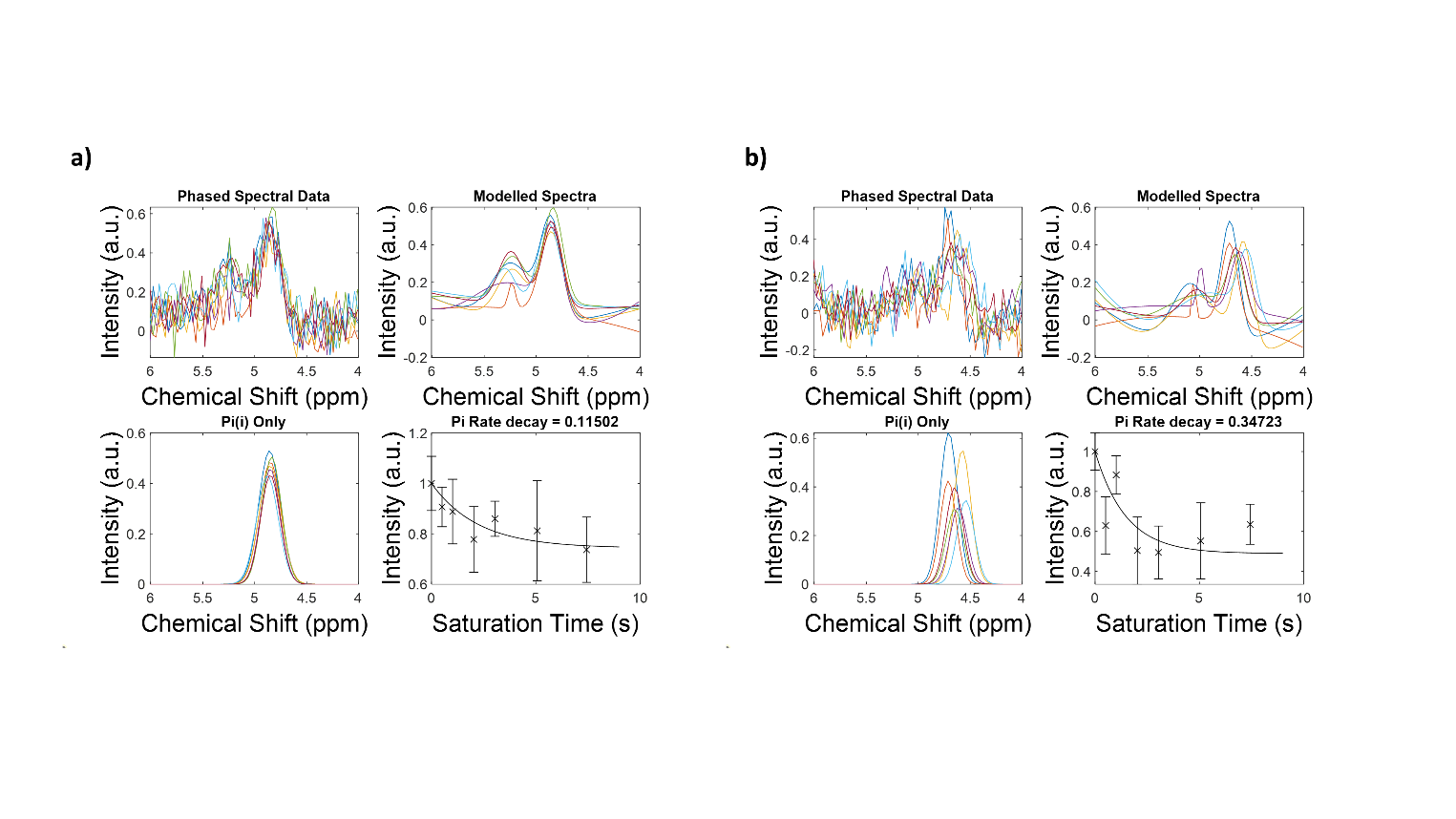


S3: ^31^P raw spectral data, processing of spectra and calculation of k rate for inorganic phosphate using the magnetisation transfer technique for Participant 4 a) pre b) post PBM treatment


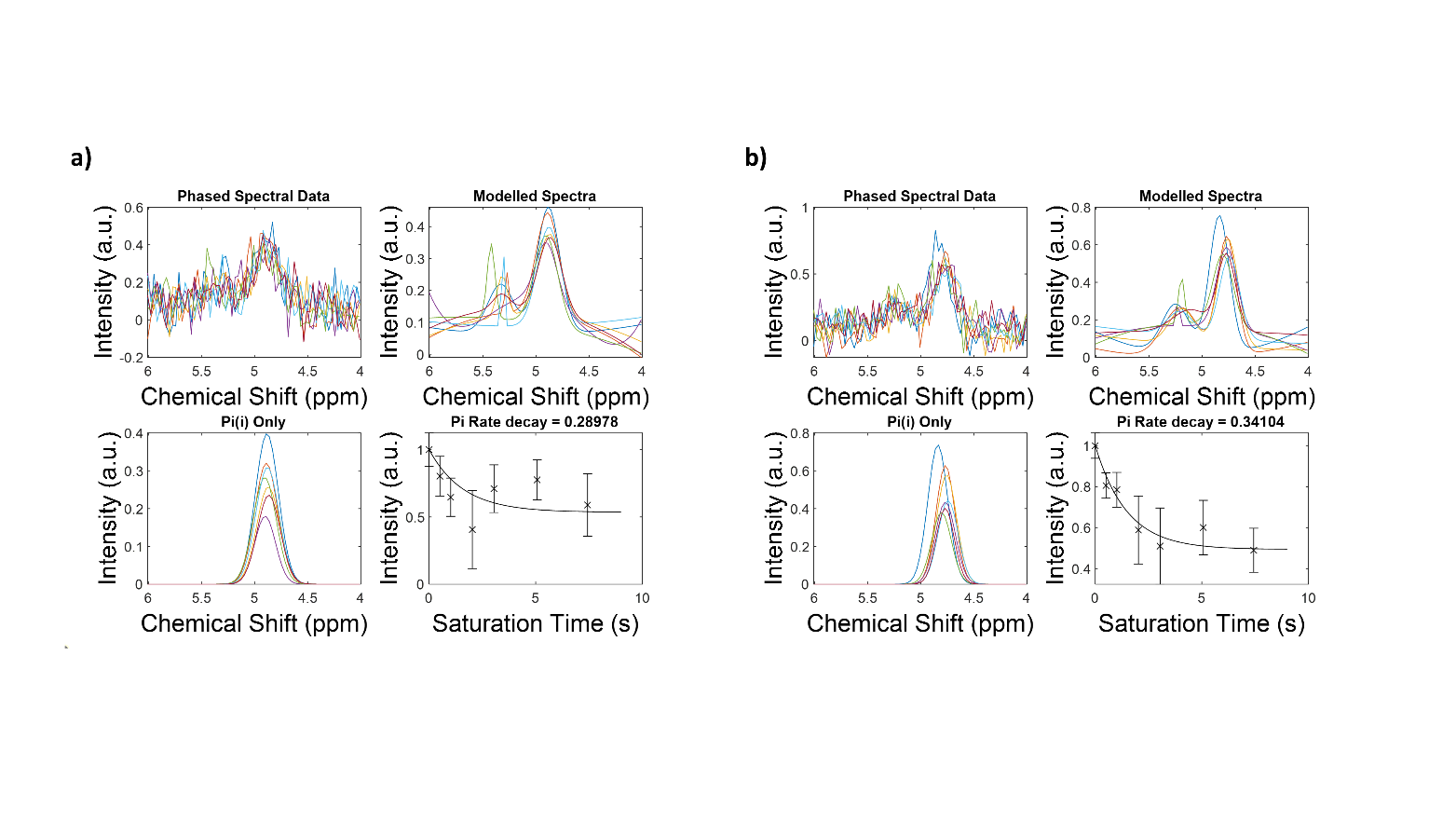


S4: ^31^P raw spectral data, processing of spectra and calculation of k rate for inorganic phosphate using the magnetisation transfer technique for Participant 5 a) pre b) post PBM treatment


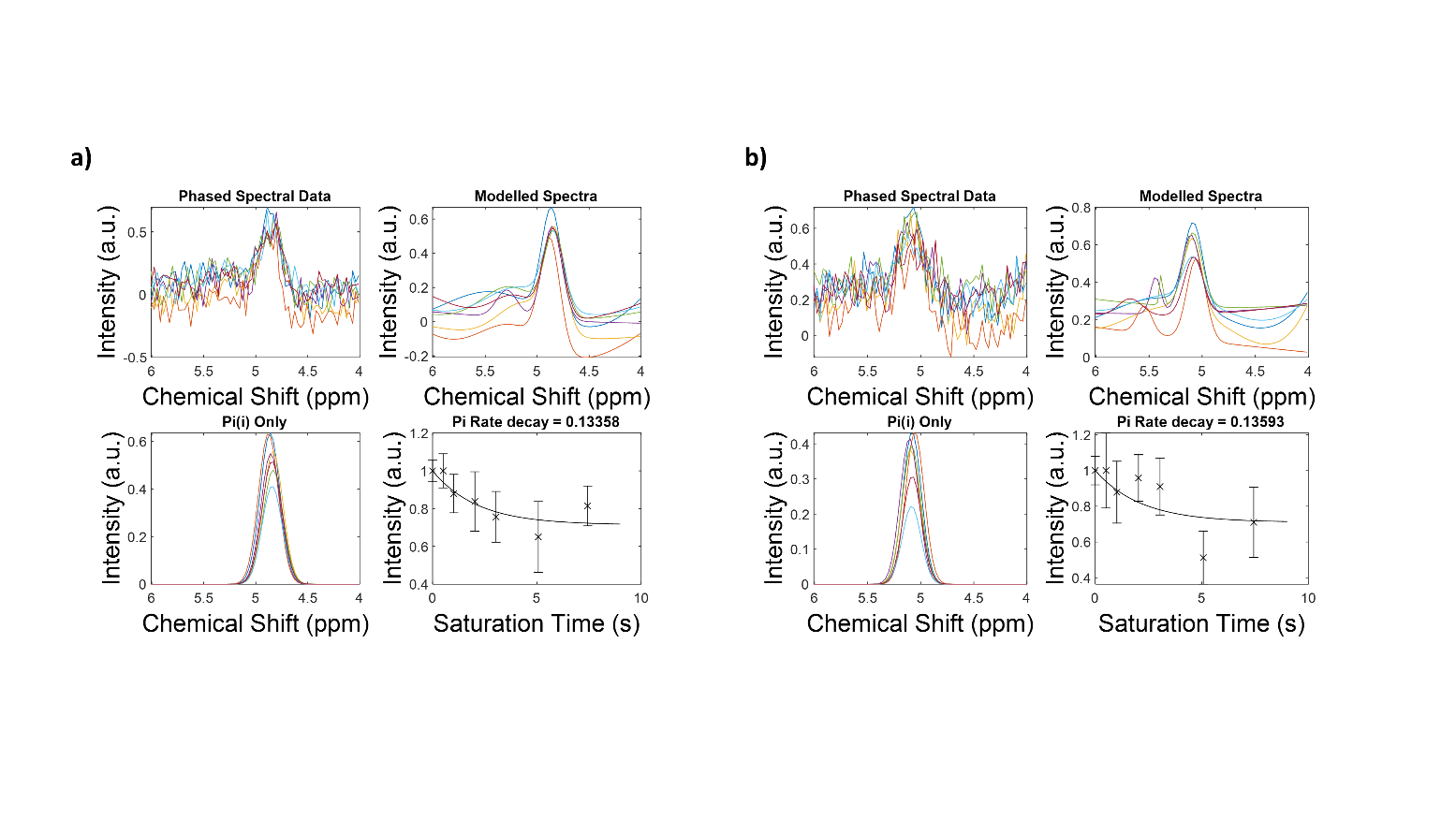


S5: ^31^P raw spectral data, processing of spectra and calculation of k rate for inorganic phosphate using the magnetisation transfer technique for Participant 7 a) pre b) post PBM treatment


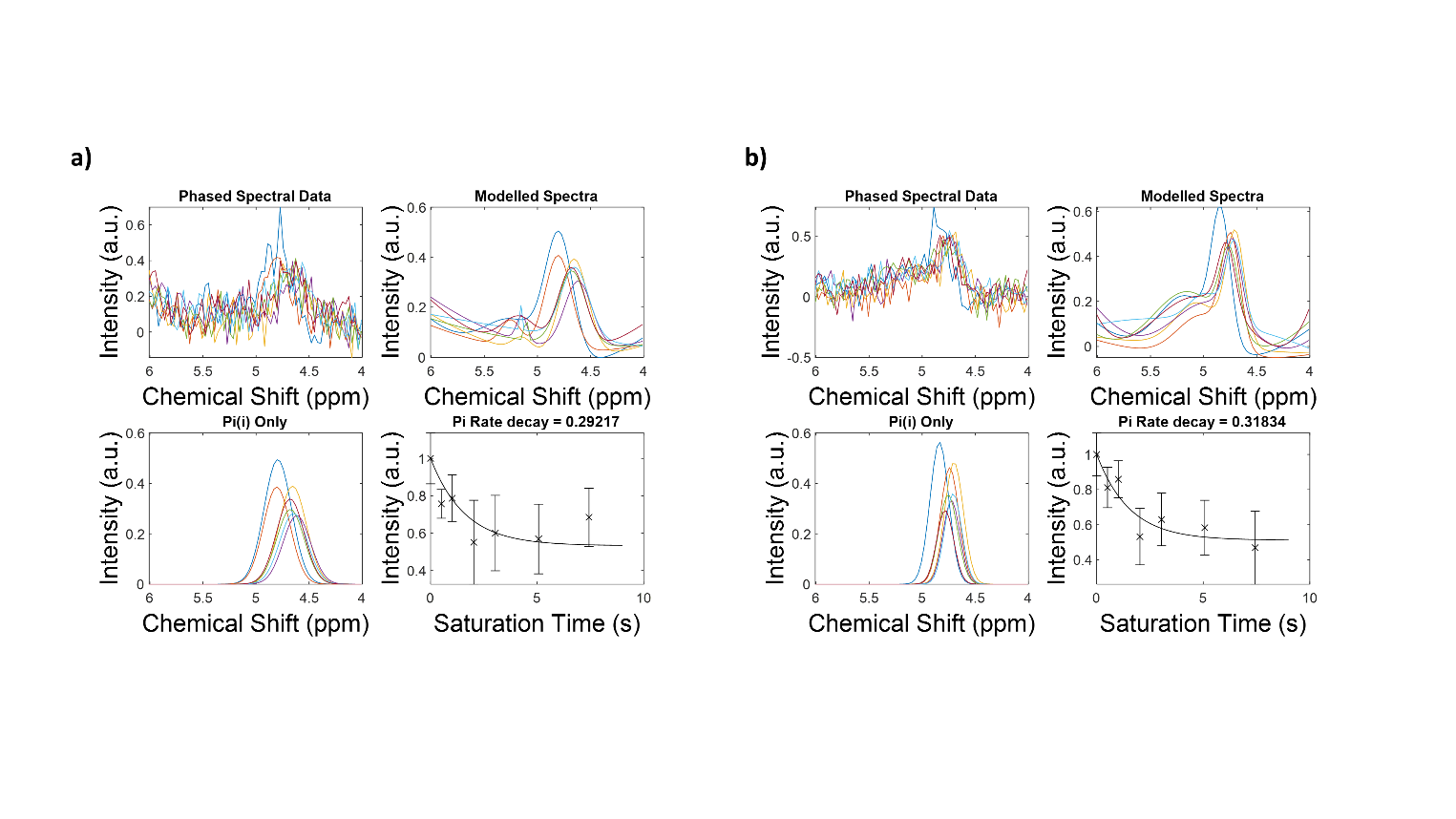


S6: ^31^P raw spectral data, processing of spectra and calculation of k rate for inorganic phosphate using the magnetisation transfer technique for Participant 9 a) pre b) post PBM treatment


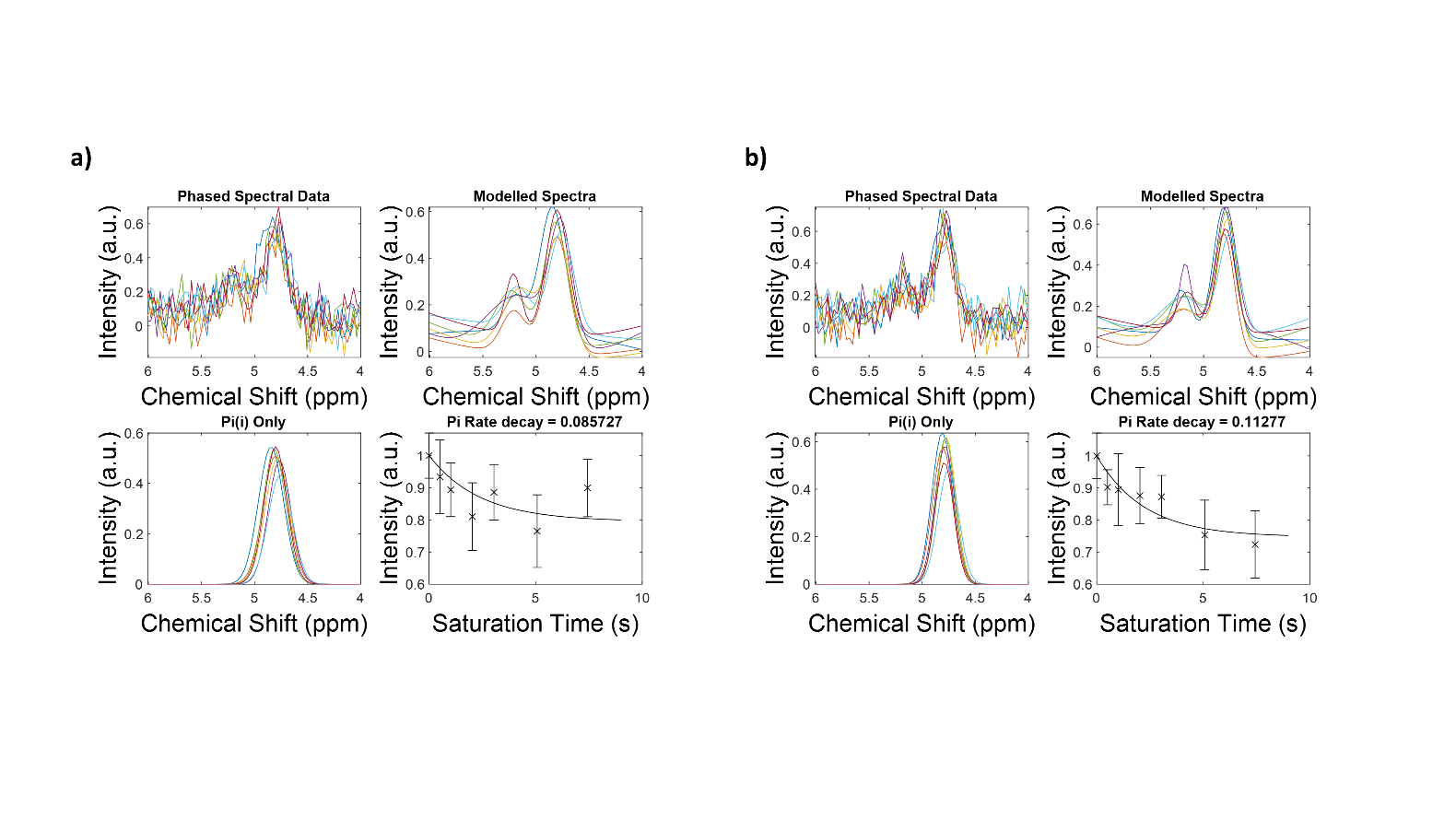


S7: ^31^P raw spectral data, processing of spectra and calculation of k rate for inorganic phosphate using the magnetisation transfer technique for Participant 10 a) pre b) post PBM treatment
